# Supplementary material for: Characterizing SARS-CoV-2 neutralization profiles after bivalent boosting using antigenic cartography
Source: Nat Commun. 2023 Aug 26;14:5224. doi: 10.1038/s41467-023-41049-4 (PMC10460376; doi:10.1038/s41467-023-41049-4)
Supplement: Supplementary file 1 — Supplementary Information [file 41467_2023_41049_MOESM1_ESM.pdf]

Supplementary Materials

Supplementary Figures

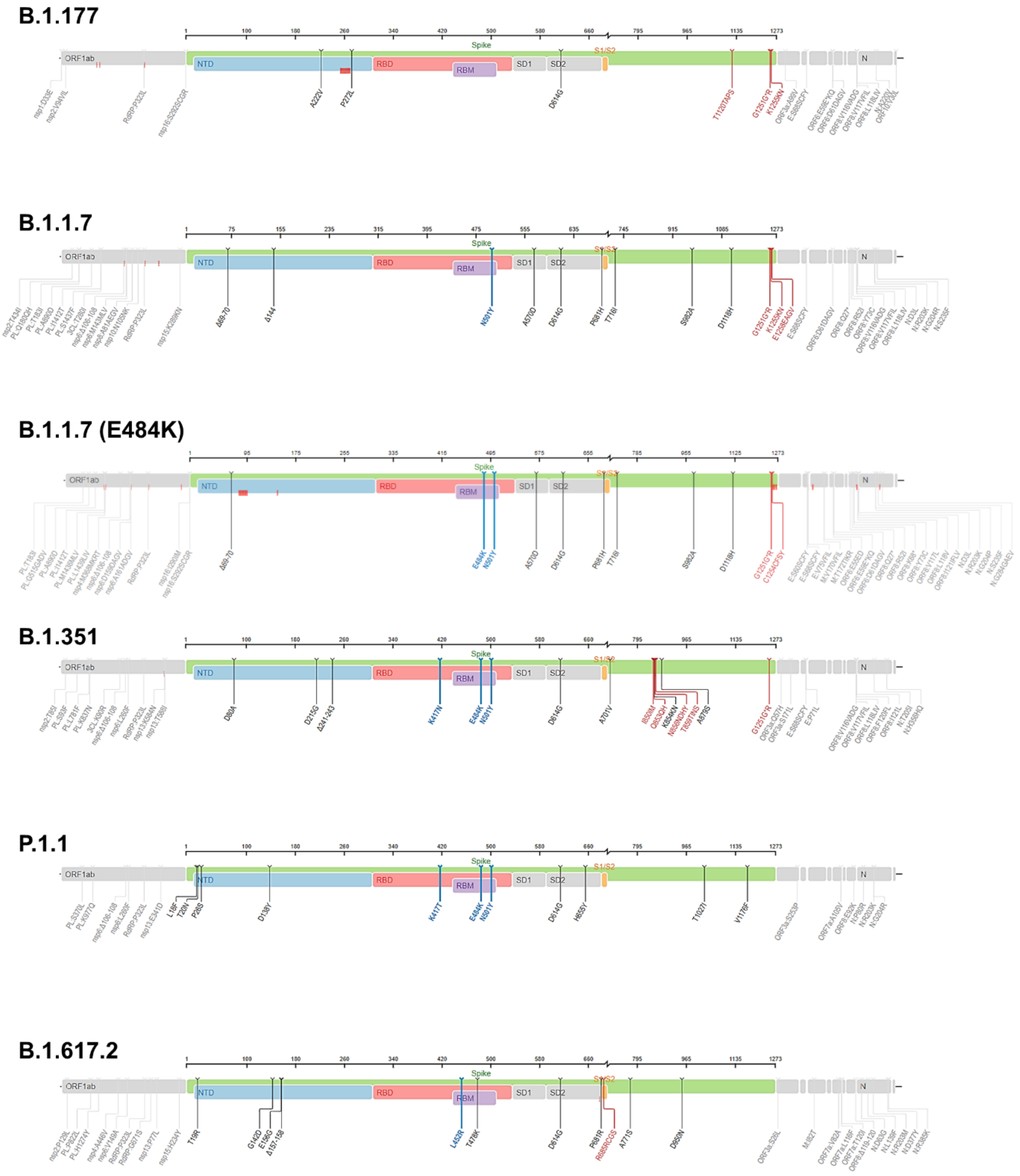

Supplementary Figure 1. Spike mutations of pre-Omicron virus isolates. Graphics depicting spike mutations in pre-Omicron variants (see Supplementary Table 2 for GISAID ID of isolated virus stocks) relative to Wuhan-1 were generated using https://covdb.stanford.edu/sierra/sars2/by-sequences/.

BA.1

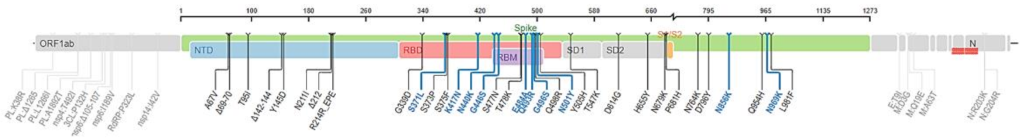

Supplementary Figure 2. Spike mutations of BA.1 Omicron virus isolate. Graphics depicting spike mutations in BA.1 Omicron variant (see Supplementary Table 2 for GISAID ID of isolated virus stock) relative to Wuhan-1 were generated using https://covdb.stanford.edu/sierra/sars2/by-sequences/.

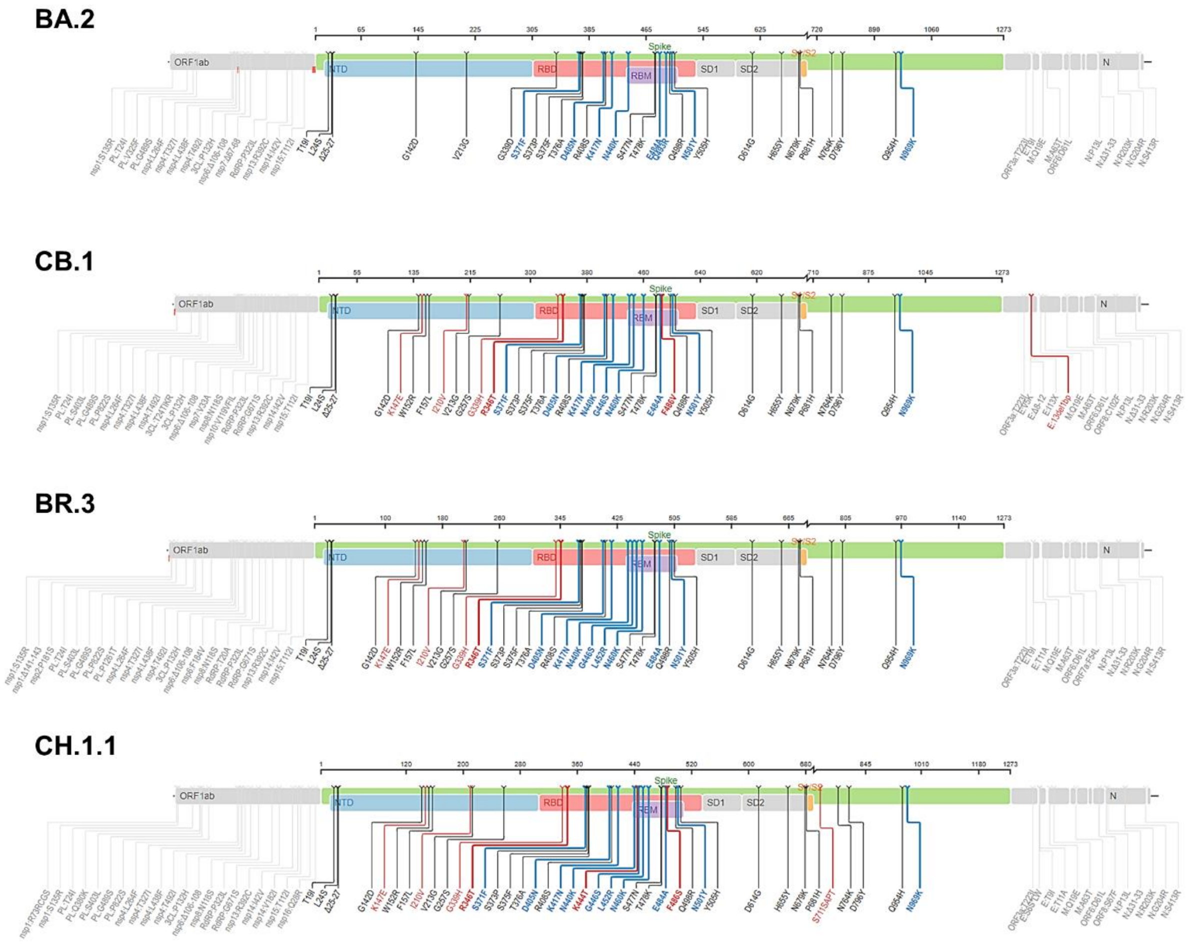

Supplementary Figure 3. Spike mutations of BA.2 Omicron virus isolates. Graphics depicting spike mutations in pre-Omicron variants (see Supplementary Table 2 for GISAID ID of isolated virus stocks) relative to Wuhan-1 were generated using https://covdb.stanford.edu/sierra/sars2/by-sequences/.

BA.5.3.2

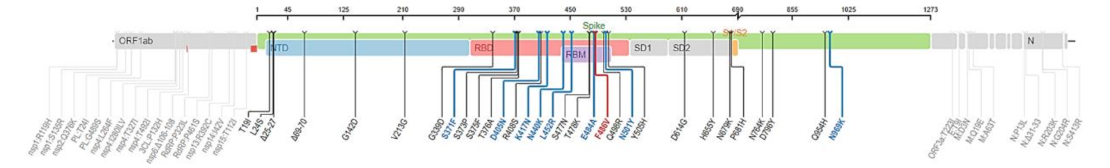

BA.5.2.1

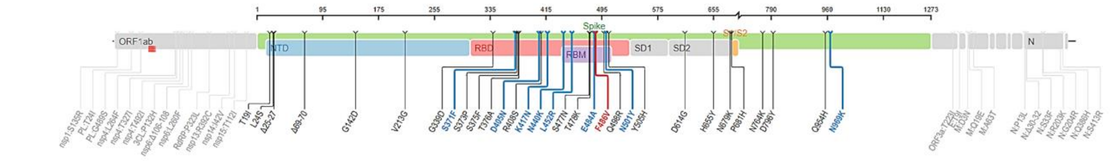

BE.1.1

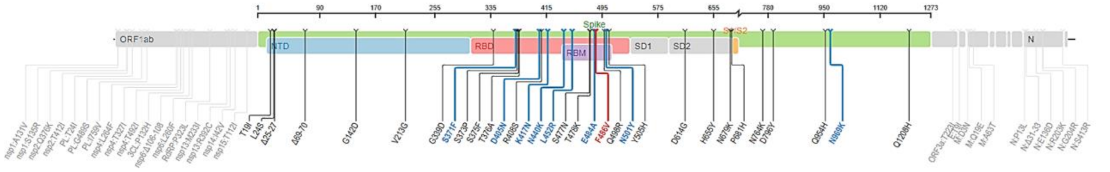

BF.7

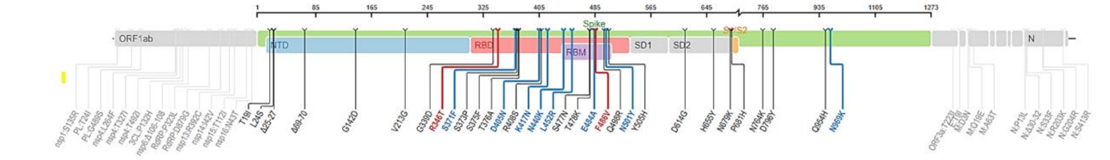

BQ.1.3

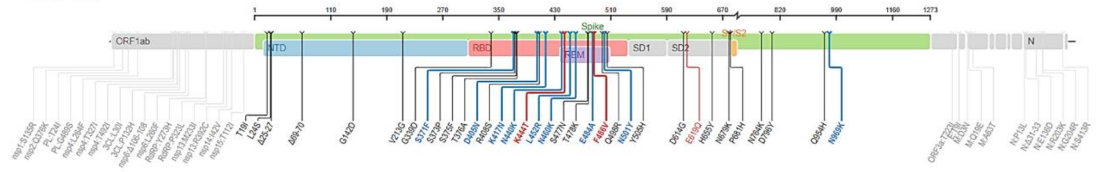

BQ.1.1

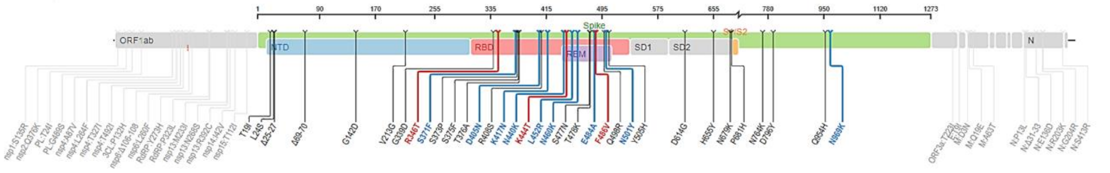

BQ.1.18

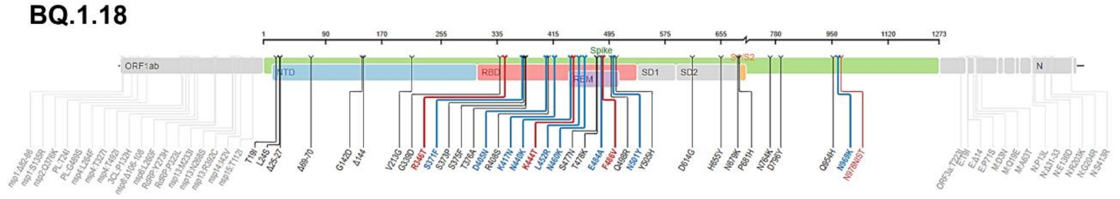

Supplementary Figure 4. Spike mutations of BA.5 Omicron virus isolates. Graphics depicting spike mutations in pre-Omicron variants (see Supplementary Table 2 for GISAID ID of isolated virus stocks) relative to Wuhan-1 were generated using https://covdb.stanford.edu/sierra/sars2/by-sequences/.

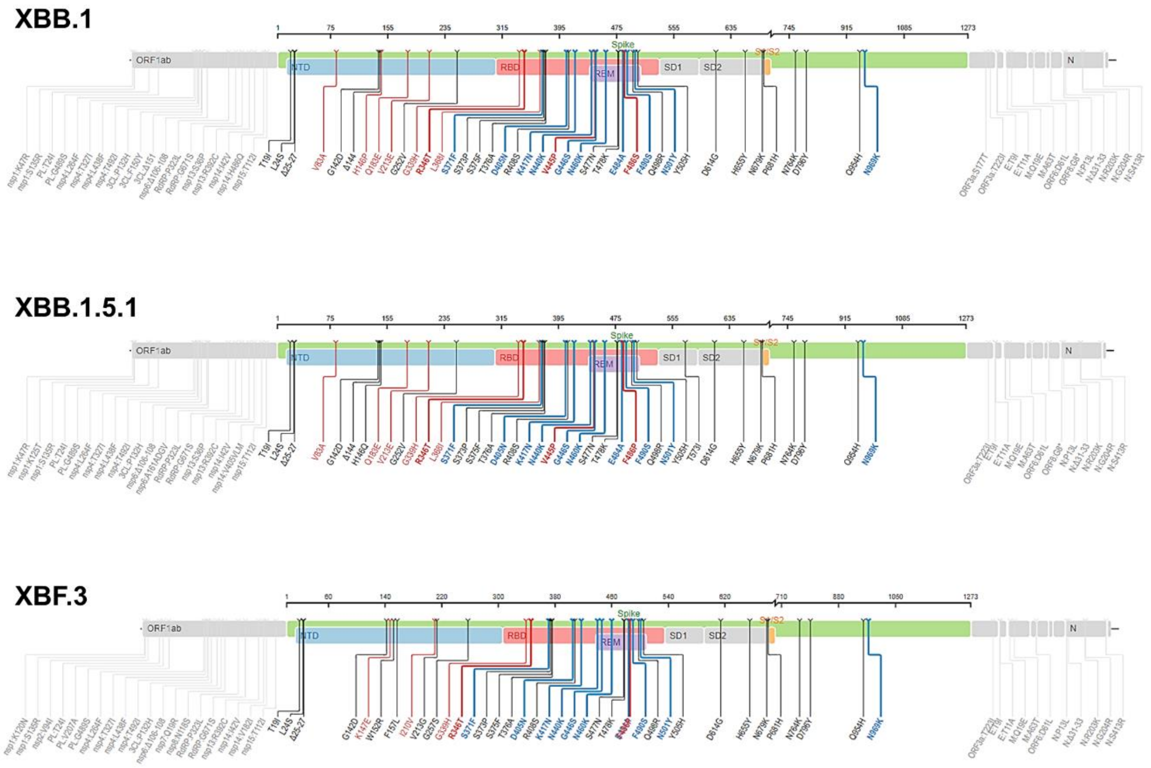

Supplementary Figure 5. Spike mutations of recombinant Omicron virus isolates. Graphics depicting spike mutations in recombinant variants (see Supplementary Table 2 for GISAID ID of isolated virus stocks) relative to Wuhan-1 were generated using https://covdb.stanford.edu/sierra/sars2/by-sequences/.

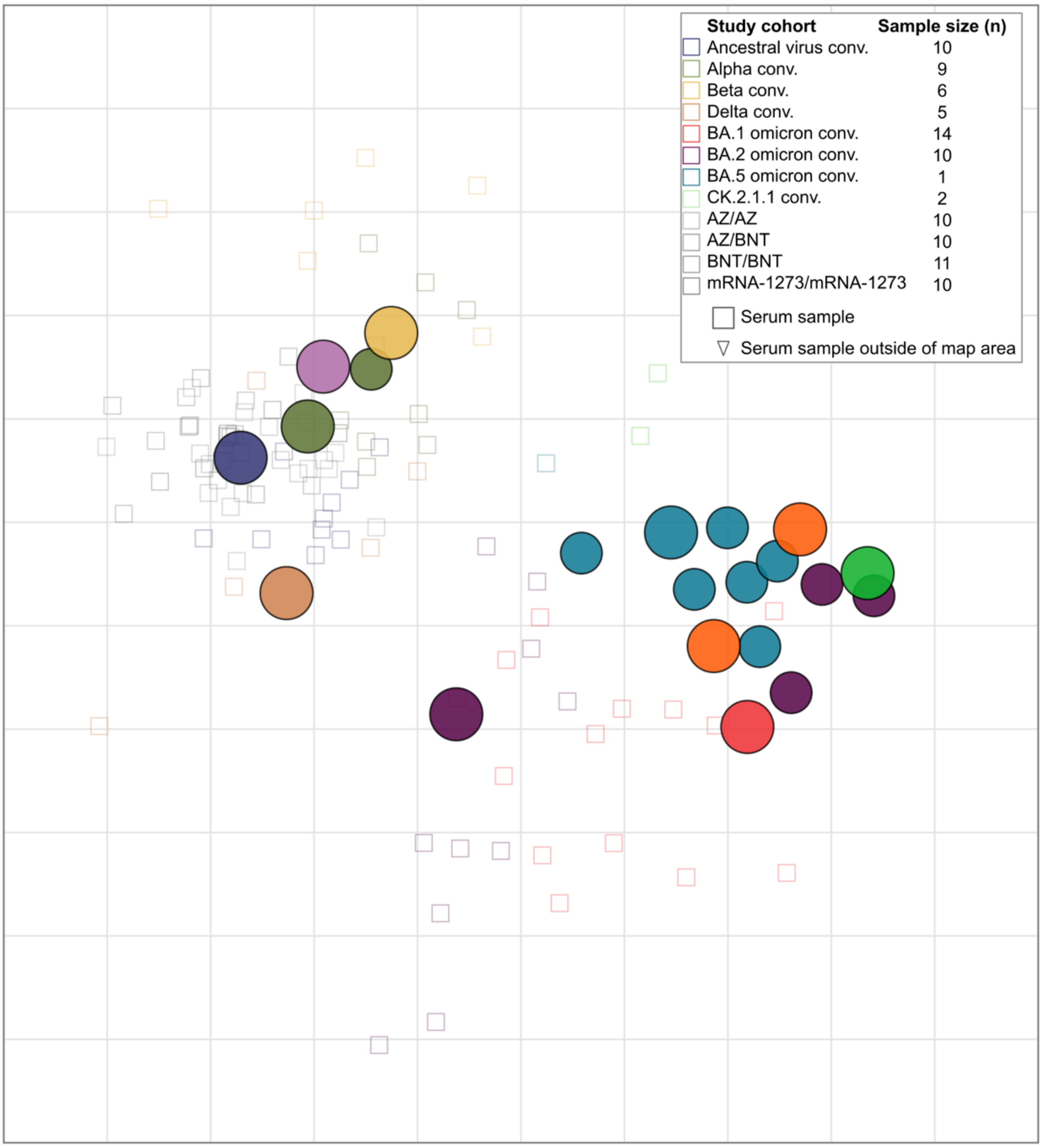

Supplementary Figure 6. Non-zoomed in version of the antigenic map. Virus variants are shown as colored circles, sera as open squares with the color corresponding to the infecting variant, vaccine sera are shown in grey tones. A smaller circle denotes variants with additional substitutions from the root variant (alpha+E484K, BA.5 and BA.2.75 sub-lineages). The x- and y-axis represent antigenic distances with one grid square corresponding to one two-fold serum dilution of the neutralization titer. The map orientation within x- and y-axis is free as only relative distances can be inferred. Only single variant exposure sera and double vaccination sera have been used for construction of the map. See Supplementary Table 3 for numbers of sera and virus variants used for calculation of the map.

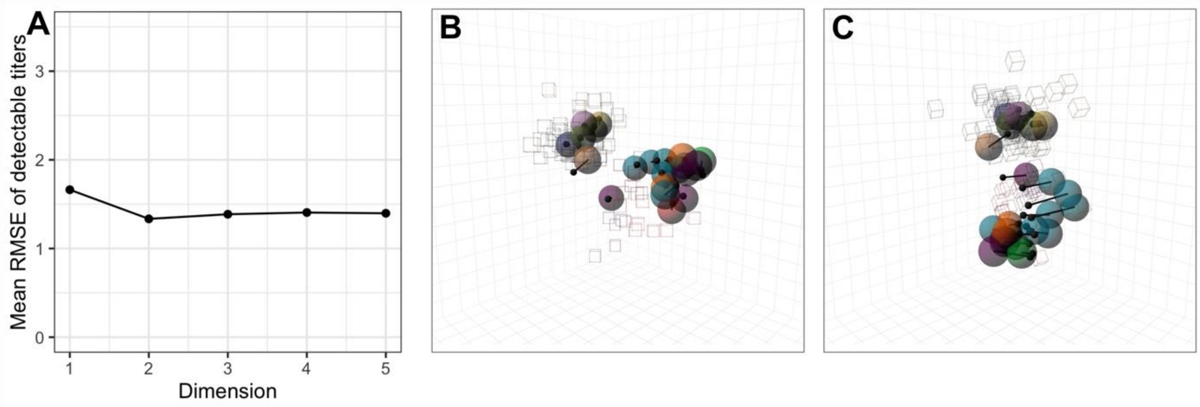

Supplementary Figure 7. Map dimensionality. (A) Dimensionality test: RMSE between map and measured titers for detectable titers in 1 to 5 dimensions. Per dimension, 100 map replicates were constructed from 90% of measured titers with 1000 optimizations per replicate. The titers of the remaining 10% were predicted in each run and the RMSE calculated by comparing the predicted to the measured titers on the $\log_2$ scale. (B, C) Side and front view of the map optimized in 3 dimensions with arrows pointing to the variants' position in the 2D map.

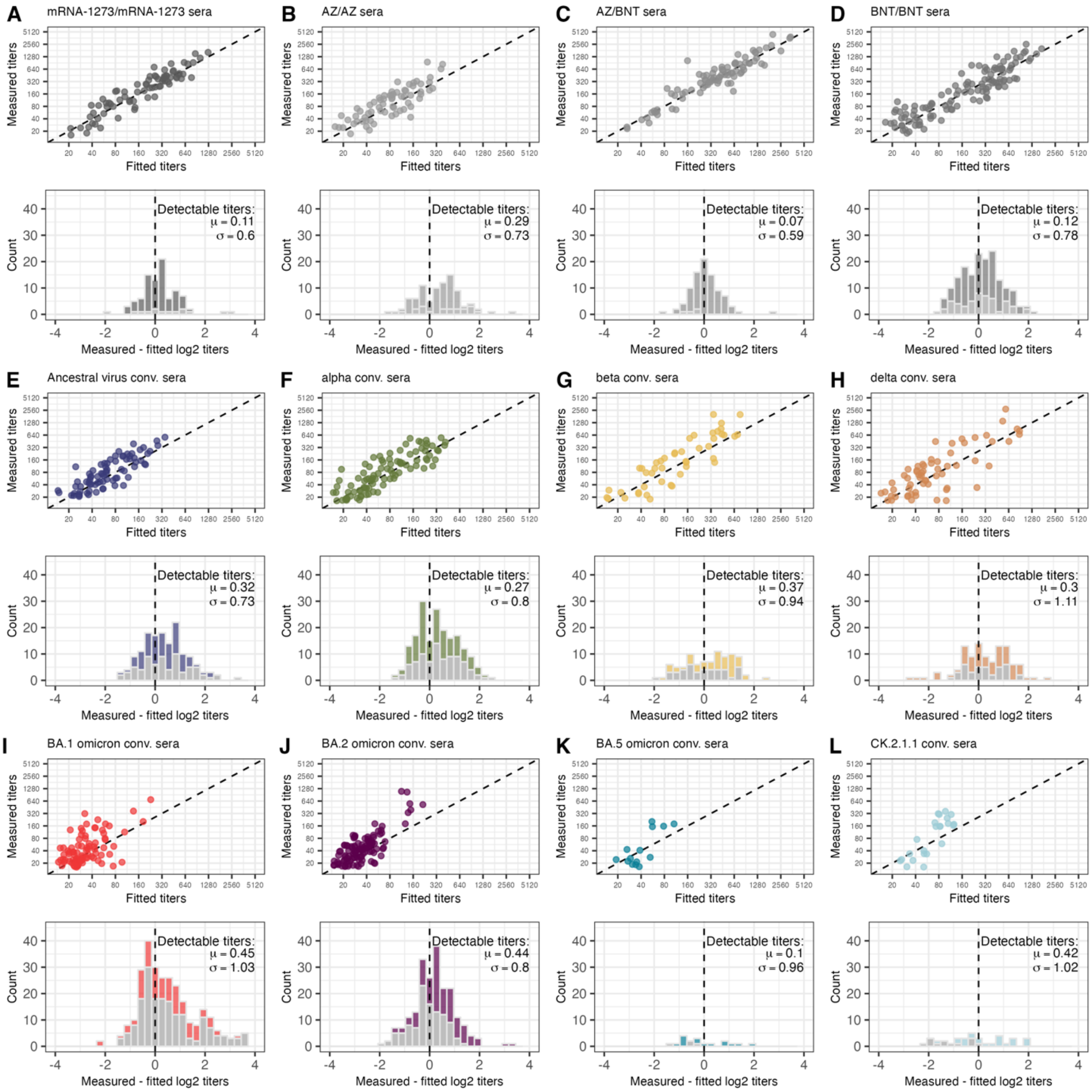

Supplementary Figure 8. Goodness of map fit per serum group. The top panels show the correlation of detectable measured and fitted titers in the 2D map with P.1.1 reactivity adjustment. Measured P.1.1 titers were reduced by one two-fold to match the reactivity adjustment in the map. Map distances were converted into $\log_2$ titers by subtracting the Euclidean distance for each serum-antigen pair from the maximum $\log_2$ titer of the specific serum. The bottom panels show the residuals of measured against fitted titers on the $\log_2$ scale, light grey marks pairs with the measured titer below the assay detection threshold. The mean and mean-centered standard deviation of differences between fitted and detectable measured titers are given in the legend of each bottom row panel. This was done for the serum groups used to construct the map: (A) mRNA-1273/mRNA-1273, (B) ChAdOx-S1/ChAdOx-S1, (C) ChAdOx-S1/BNT162b2, (D) BNT162b2/BNT162b2, (E) Ancestral virus conv., (F) alpha/alpha+E484K conv., (G) beta conv., (H) delta conv., (I) BA.1 Omicron conv., (J) BA.2 Omicron conv., (K) BA.5 Omicron conv., (L) CK.2.1.1 conv..

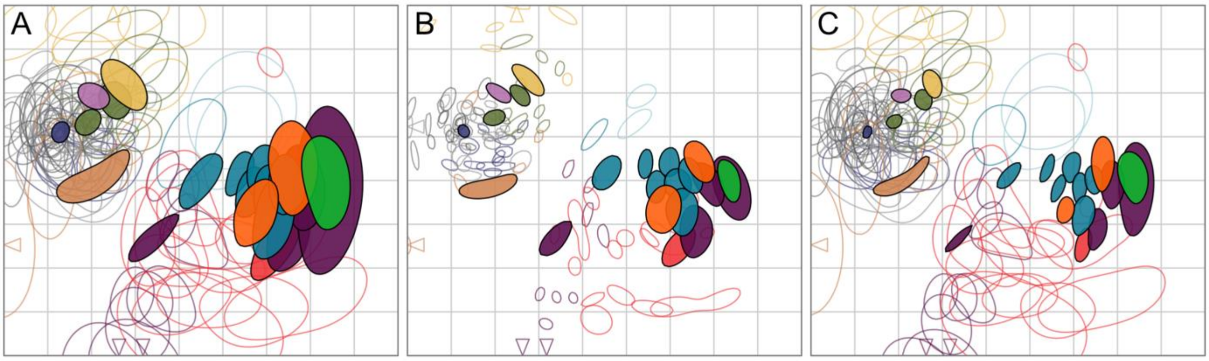

Supplementary Figure 9. Assessing map robustness by bootstrapping. 500 bootstrap repeats were performed with 1000 optimizations per repeat. In each repeat, different weights are assigned to each part of the titer table. The weights are drawn randomly from a Dirichlet distribution. Different weights were added to serum and antigen reactivity (A), only serum reactivity (B), and only antigen reactivity (C). The colored regions mark 68% (one standard deviation) of the positional variation for each variant (filled shapes) and sera (open shapes). The colors correspond to the colors used in Figure 2.

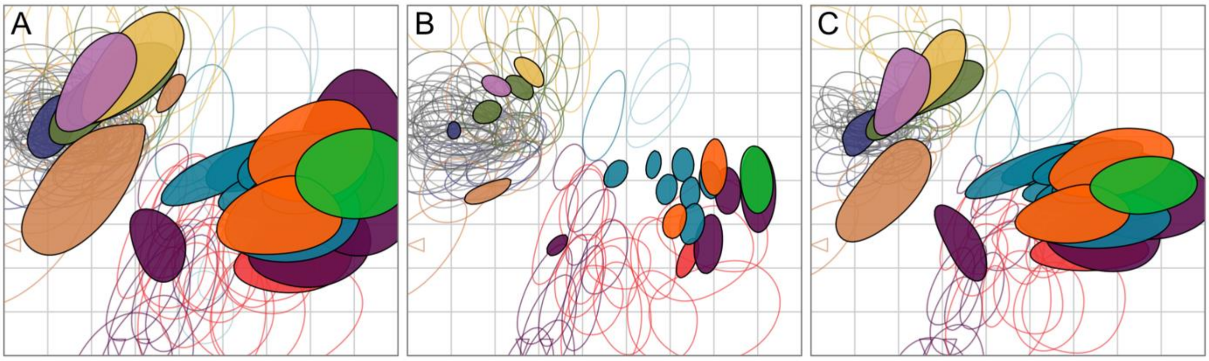

Supplementary Figure 10. Assessing map robustness to measurement uncertainty by bootstrapping. 500 bootstrap repeats were performed with 1000 optimizations per repeat. Normally distributed measurement noise with a standard deviation of 0.7 was added to titers and antigen reactivity (A), only titers (B), and only antigen reactivity (C). The colored regions mark 68% (one standard deviation) of the positional variation for each variant (filled shapes) and sera (open shapes). The colors correspond to the colors used in Figure 2.

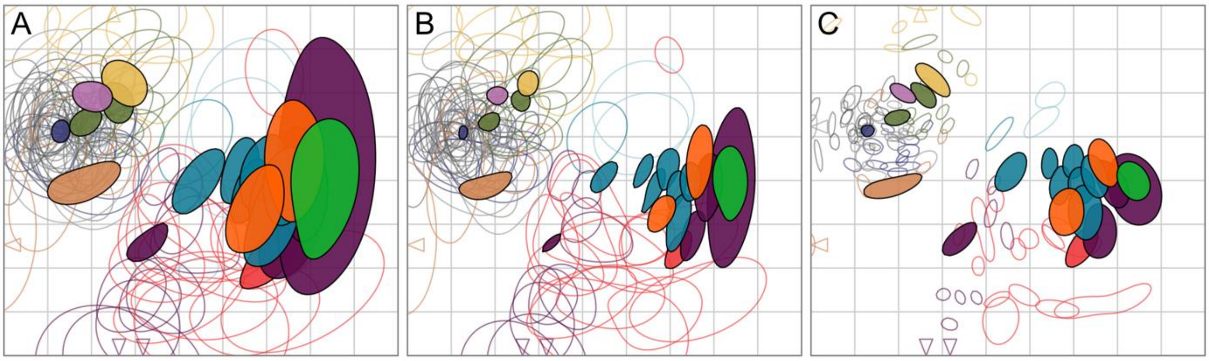

Supplementary Figure 11. Assessing map robustness to the exclusion of measurements by bootstrapping. 500 bootstrap repeats were performed with 1000 optimizations per repeat. For each repeat, a random subset of titer measurements was taken with replacement. The bootstrapping was performed on variants and sera (A), only variants (B), and only sera (C). The colored regions mark 68% (one standard deviation) of the positional variation for each variant (filled shapes) and sera (open shapes). The colors correspond to the colors used in Figure 2.

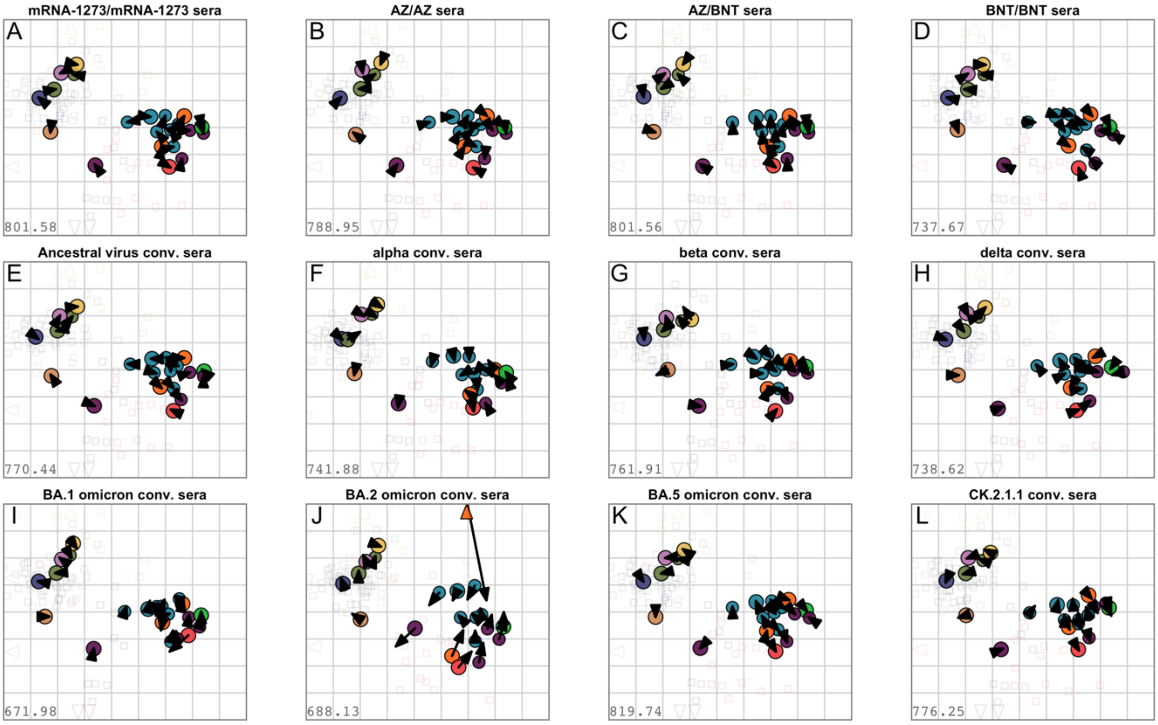

Supplementary Figure 12. Assessing map robustness to the exclusion of sera. Each serum group was removed and the map reoptimized. Arrows point to the position of each variant in the map shown in Figure X, for color correspondence refer to this map. A small arrow length indicates similar variant positions and map robustness to the exclusion of the particular serum group. Triangles point to sera positioned outside the plotting area. Maps without mRNA-1273/mRNA-1273 (A), ChAdOx-S1/ChAdOx-S1 (B), ChAdOx-S1/BNT162b2 (C), BNT162b2/BNT162b2 (D), Ancestral virus conv. (E), alpha/alpha+E484K conv. (F), beta conv. (G), delta conv. (H), BA.1 Omicron conv. (I), BA.2 Omicron conv. (J), BA.5 Omicron conv. (K), CK.2.1.1 conv. (L).

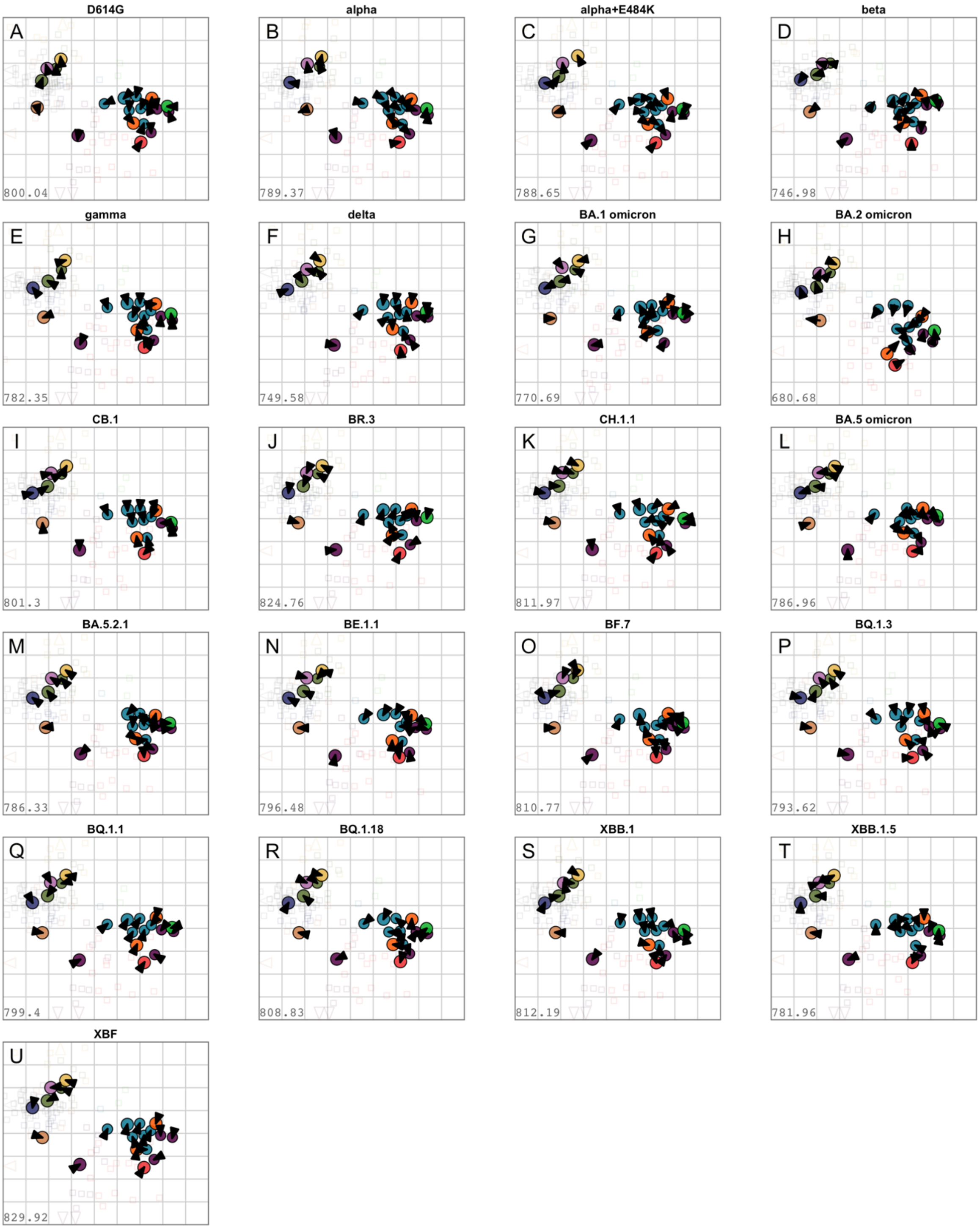

Supplementary Figure 13. Assessing map robustness to the exclusion of antigen variant. Each antigen variant was removed and the map reoptimized. Arrows point to the position of each variant in the map shown in Figure 3, for color correspondence refer to this map. A small arrow length indicates similar variant positions and robustness to the exclusion of the particular antigen variant. Triangles point to sera positioned outside the plotting area. Maps without D614G (A), alpha (B), alpha+E484K (C), gamma (D), beta (E), delta (F), BA.1 Omicron (G), BA.2 Omicron (H), CB.1 (I), BR.3 (J), CH.1.1 (K), BA.5 Omicron (L), BA.5.2.1 (M), BE.1.1 (N), BF.7 (O), BQ.1.3 (P), BQ.1.1 (Q), BQ.1.18 (R), XBB.1 (S), XBB.1.5 (T), XBF (U).

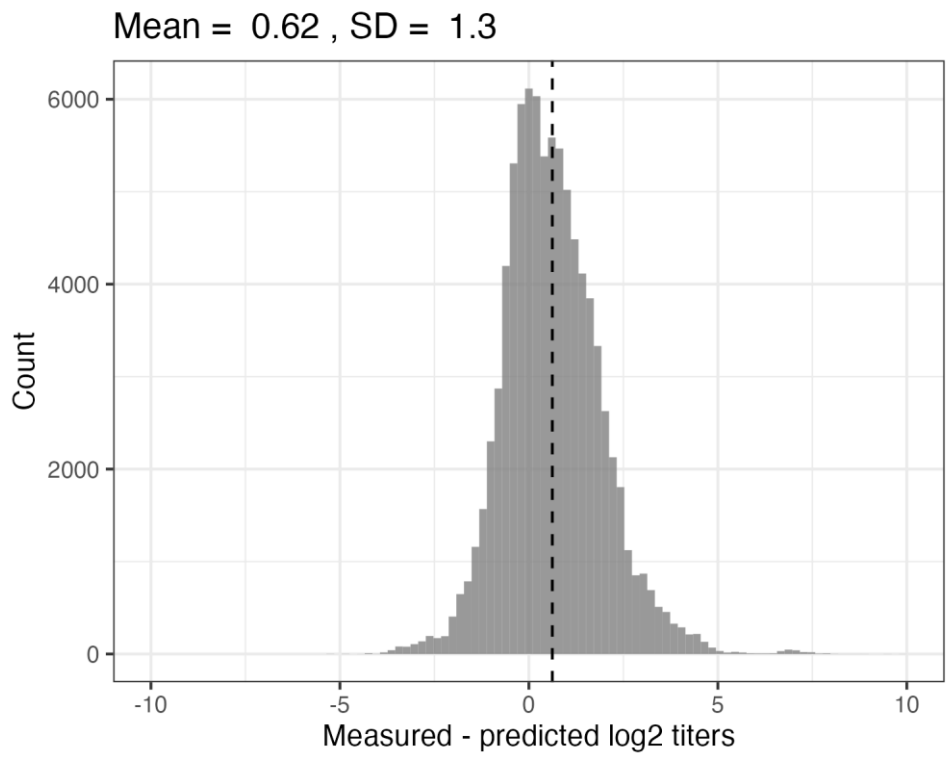

Supplementary Figure 14. Map cross-validation residual titers. 1000 repeats with 1000 optimization runs each were performed with only 90% of measured titers used for map construction by artificially masking 10% of measurements. The missing $\log_2$ titers were predicted by subtracting the Euclidean map distance for each serum-antigen pair from the maximum $\log_2$ titer of the specific serum. The difference between predicted and detectable measured titers on the $\log_2$ scale was calculated, the mean is indicated by the dashed line. The mean and mean-centered standard deviation (SD) are given.

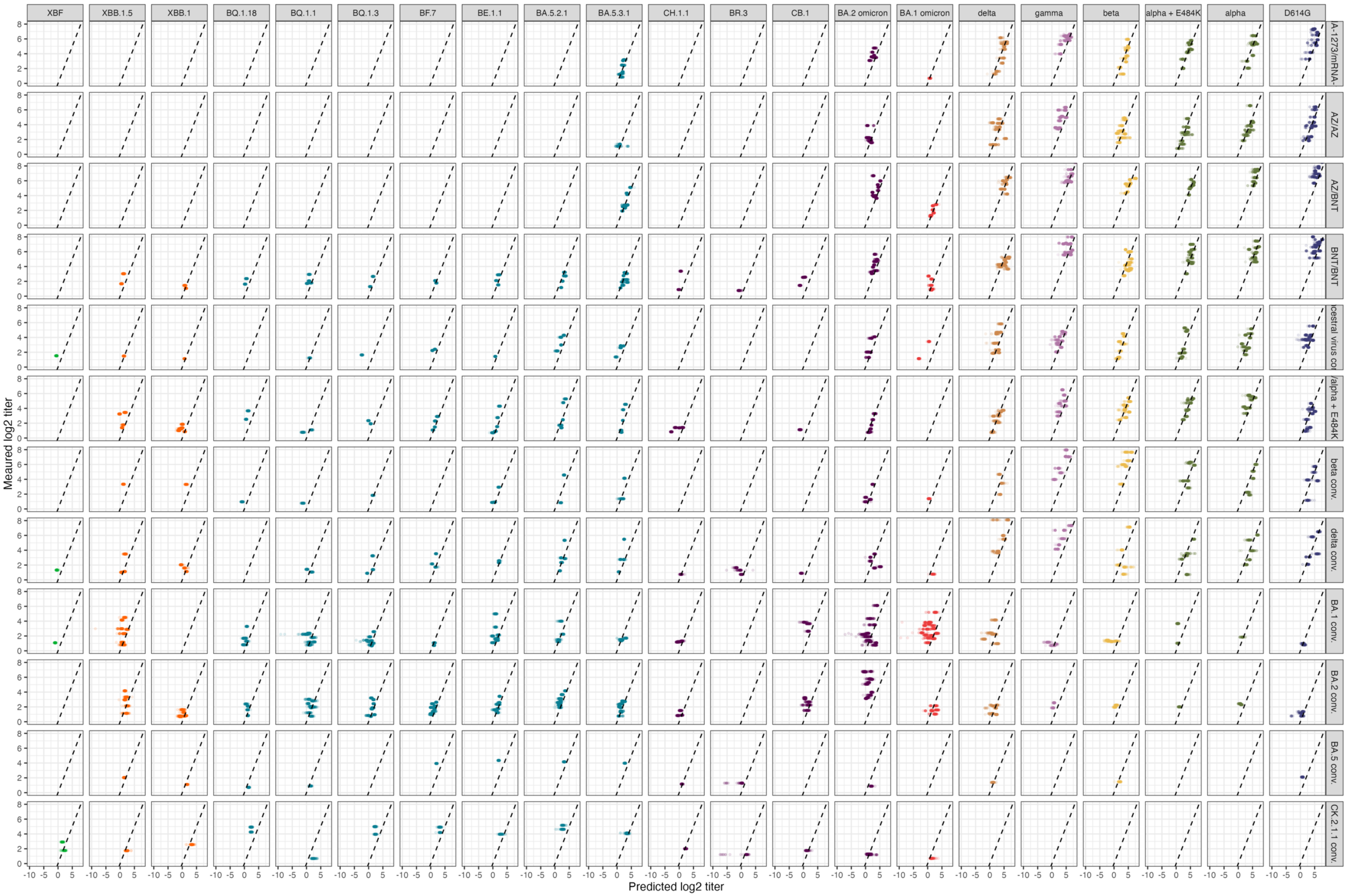

Supplementary Figure 15: Map cross-validation predicted vs. measured titers. Figure caption on next page.

Supplementary Figure 15. Map cross-validation predicted vs. measured titers. 1000 repeats with 1000 optimization runs each were performed with only 90% of measured titers used for map construction by artificially masking 10% of measurements. The missing $\log_2$ titers were predicted by subtracting the Euclidean map distance for each serum-antigen pair from the maximum $\log_2$ titer of the specific serum. The detectable measured over predicted log2 titers are shown per serum group and antigen variant. The lower x-axis limit has been set to -10 for plotting purposes, very few residuals in the Omicron convalescent groups were larger than that due to inaccurate positioning of sera.

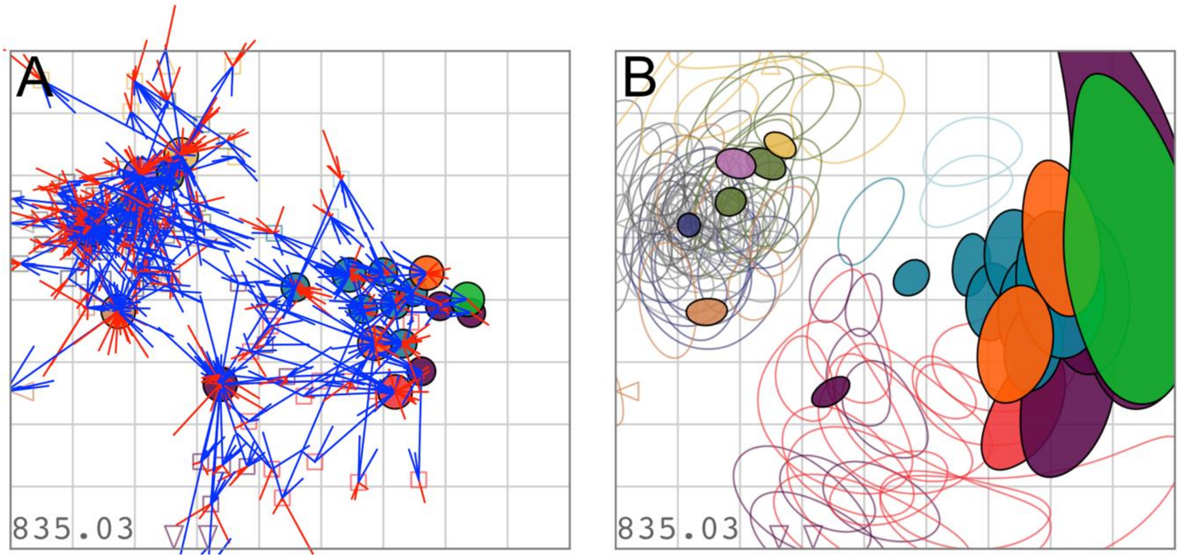

Supplementary Figure 16. Titer error lines and map triangulation. (A) Error lines for each serum and antigen are shown in blue in case of larger map distance than target distance and red in case of smaller map distance than target distance. The length of each error bar indicates the magnitude of mismatch. Blue error lines point towards the variant-serum pair that has a smaller target distance, red error lines point away from the variant-serum pair. (B) Constant force loci (Triangulation blobs) show the area for each serum and variant in which the item can move without increasing map stress by more than one unit. Filled shapes show variant Triangulation blobs, open shapes sera. Colors correspond to the map shown in Figure 2.

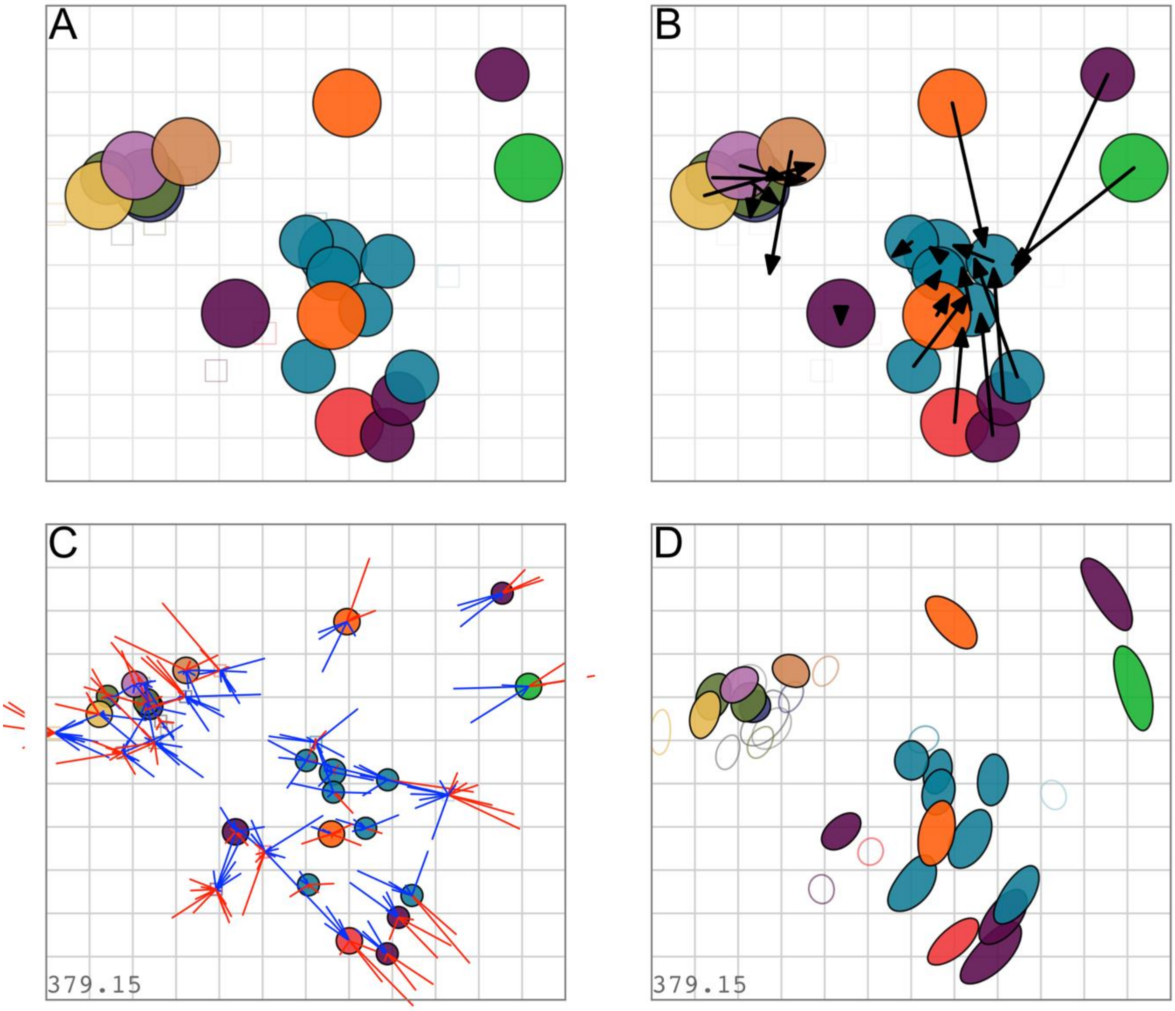

Supplementary Figure 17. Map from geometric mean titers GMT. (A) Geometric mean titers (GMT) of each serum groups were used for map construction. The colors correspond to the colors used in Figure 2. (B) Map in A with arrows pointing towards the variants' positions in Figure 2. (C) Error lines connecting GMT sample and variant. Blue lines indicate a larger map than target distance, red lines indicate a smaller map than target distance. (D) Constant force foci (Triangulation blobs) of variants and GMT samples. The marked area corresponds to the area an item can occupy without increasing the map stress by more than 1 unit. Filled shapes show variant Triangulation blobs, open shapes sera.

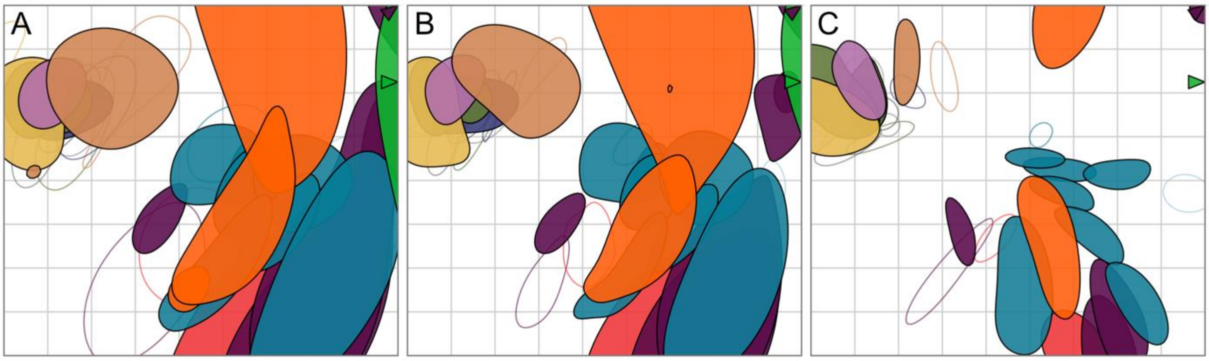

Supplementary Figure 18. Assessing geometric mean titer (GMT) map robustness to measurement uncertainty by bootstrapping. 500 bootstrap repeats were performed with 1000 optimizations per repeat. In each repeat, different weights are assigned to each part of the titer table. The weights are drawn randomly from a Dirichlet distribution. Different weights were added to serum and antigen reactivity (A), only serum reactivity (B), and only antigen reactivity (C). The colored regions mark 68% (one standard deviation) of the positional variation for each variant (filled shapes) and sera (open shapes). The colors correspond to the colors used in Figure 2.

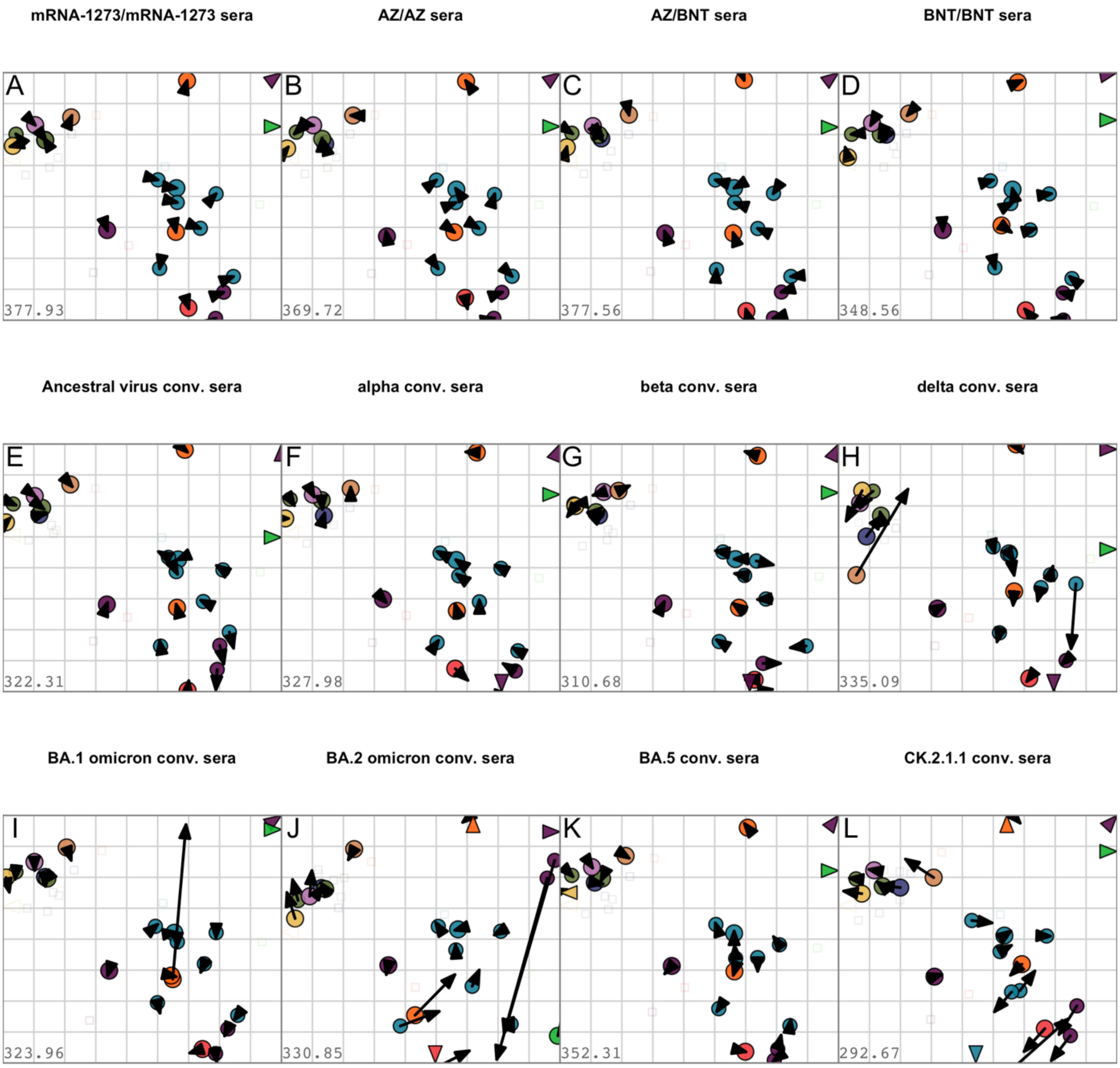

Supplementary Figure 19. Assessing geometric mean titer (GMT) map robustness to the exclusion of sera. Each serum group was removed and the map re-optimized with optimization 1000 iterations. Arrows point to the position of each variant in the GMT map shown in Supplementary Figure 17A, for color correspondence refer to the map in the main manuscript. A small arrow length indicates similar variant positions and map robustness to the exclusion of the particular serum group. Maps without mRNA-1273/mRNA-1273 (A), ChAdOx-S1/ChAdOx-S1 (B), ChAdOx-S1/BNT162b2 (C), BNT162b2/BNT162b2 (D), Ancestral virus conv. (E), alpha/alpha+E484K conv.(F), beta conv. (G), delta conv. (H), BA.1 Omicron conv. (I), BA.2 Omicron conv. (J), BA.5 Omicron conv. (K), CK.2.1.1 conv. (L).

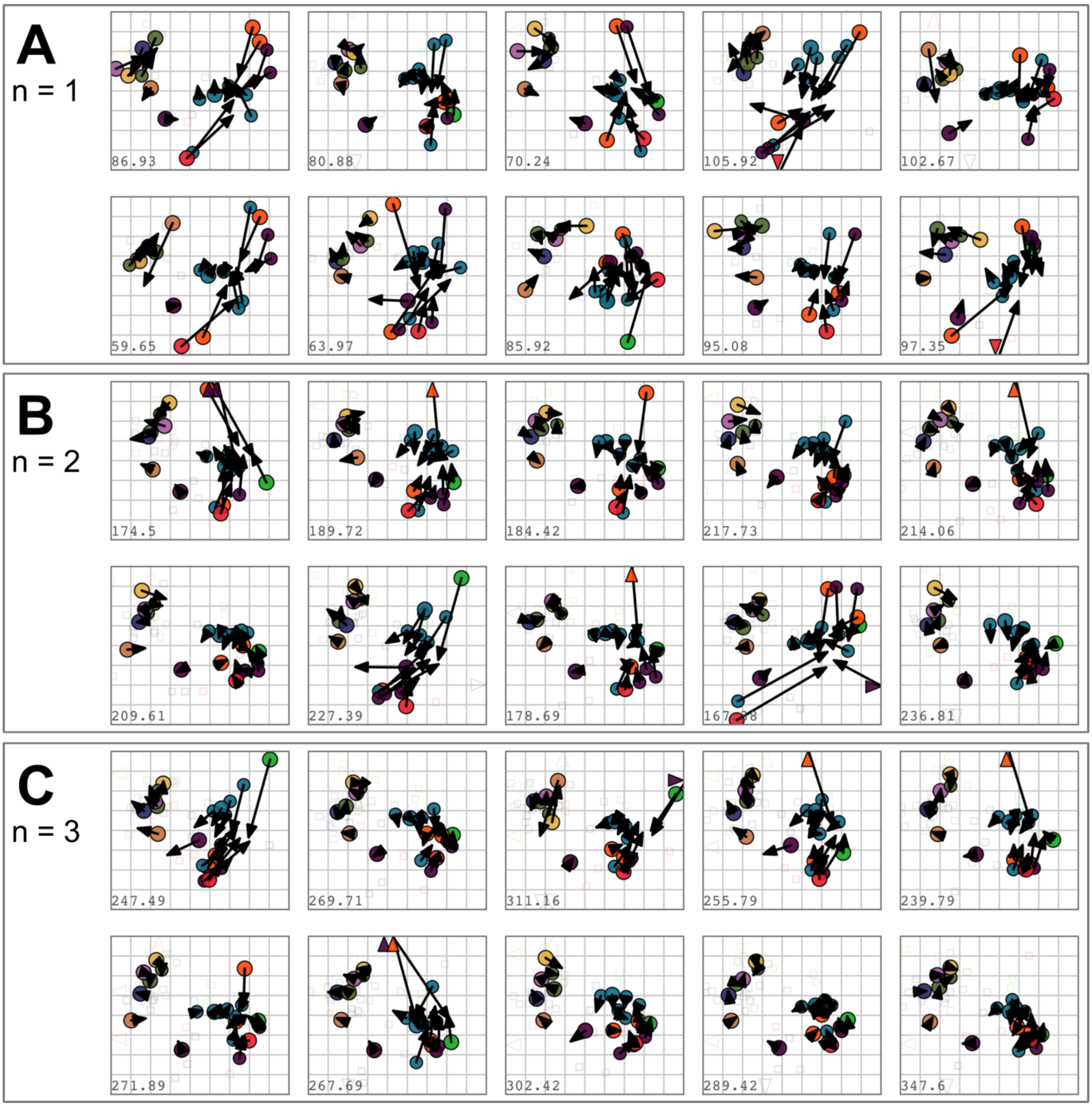

Supplementary Figure 20. Assessing map robustness to sample size per serum group. Ten Maps were created with a randomly drawn subset of samples with different sample sizes. 1000 optimizations were performed per map with a dilution step size of 0 and the minimum column basis set to “none”. (A) n=1, (B) n=2, (C) n=3 samples were randomly drawn per serum group.

A.

B.

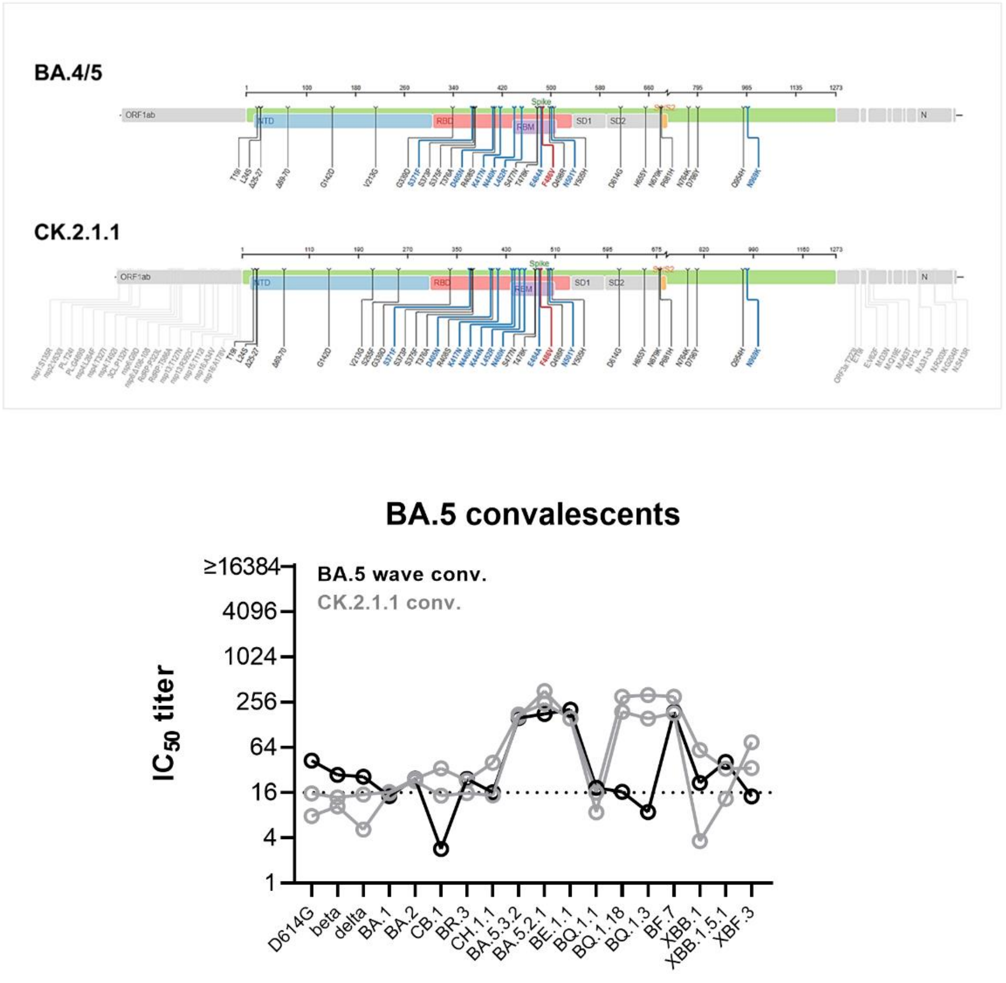

Supplementary Figure 21. Neutralization profile of BA.5 Omicron convalescent individuals. (A) Spike mutations in BA.5 compared to CK.2.1.1, generated using https://covdb.stanford.edu/sierra/sars2/by-sequences/. (B) Neutralizing antibody titers against D614G, beta, delta, BA.1, BA.2, CB.1, BR.3, CH.1.1, BA.5.3.2, BA.5.2.1, BE.1.1, BF.7, BQ.1.3, BQ.1.8, BQ.1.1, XBB.1, XBB.1.5.1, and XBF.3 variants for one BA.5 first exposure (black) and two CK.2.1.1 first exposure sera (gray) are shown. Values for each patient are connected by a line. Titers below 16 were treated as negative (dotted line). IC_{50} titer = 50% neutralization titer.

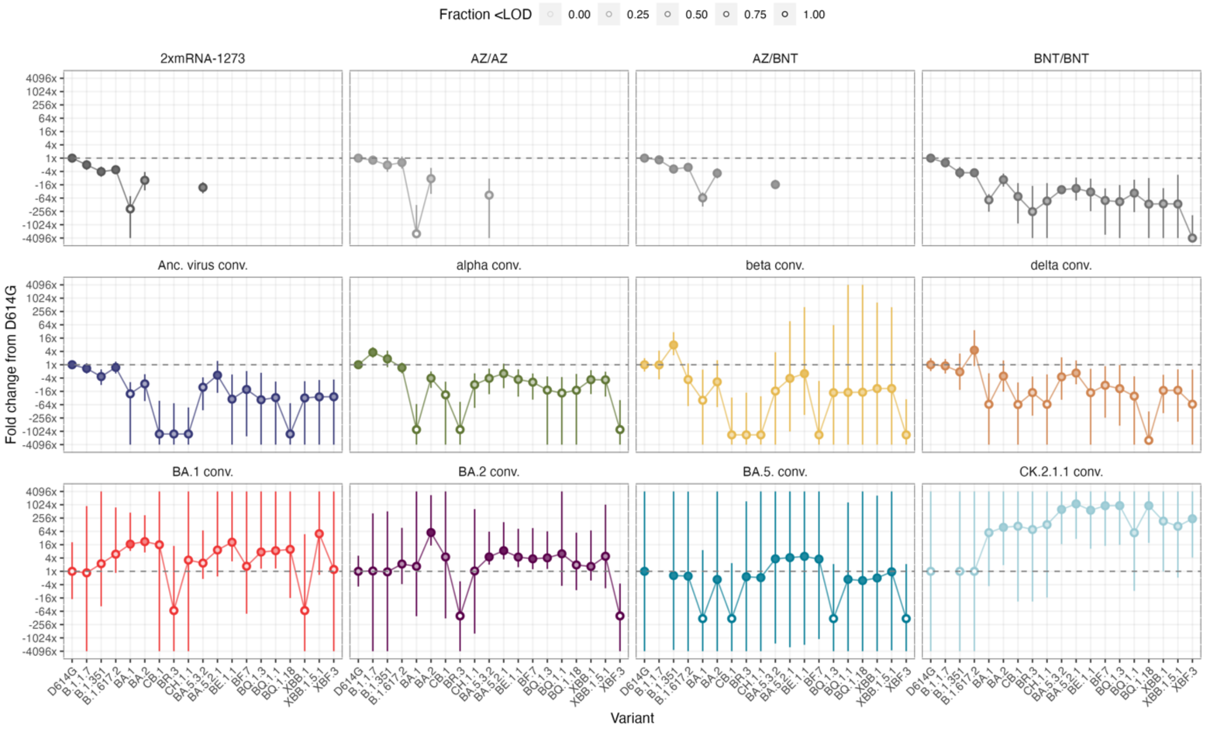

Supplementary Figure 22. Titer fold changes from D614G for map serum groups. Mean fold changes from D614G and 95% CI (confidence interval) were calculated for each map serum group (2xmRNA-1273 n= 10, AZ/AZ n=10, AZ/BNT n=10, BNT/BNT n=6-11, Anc. virus conv. n=5-10, alpha conv. n=10, beta conv. n=3-6, delta conv. n=7, BA.1 conv. n=18, BA.2 conv. n=10, BA.5 conv. n=1, CK.2.1.1 conv. n=2) using the titertools R package[1], where below threshold values are interpolated using a Bayesian approach. The whiteness of each point corresponds to the fraction of titers <LOD (limit of detection), increasing with the number of samples <LOD. For fully colored circles, all samples had detectable titers against the respective variant. Part of the data used for calculation of fold changes have been published previously in [2-4].

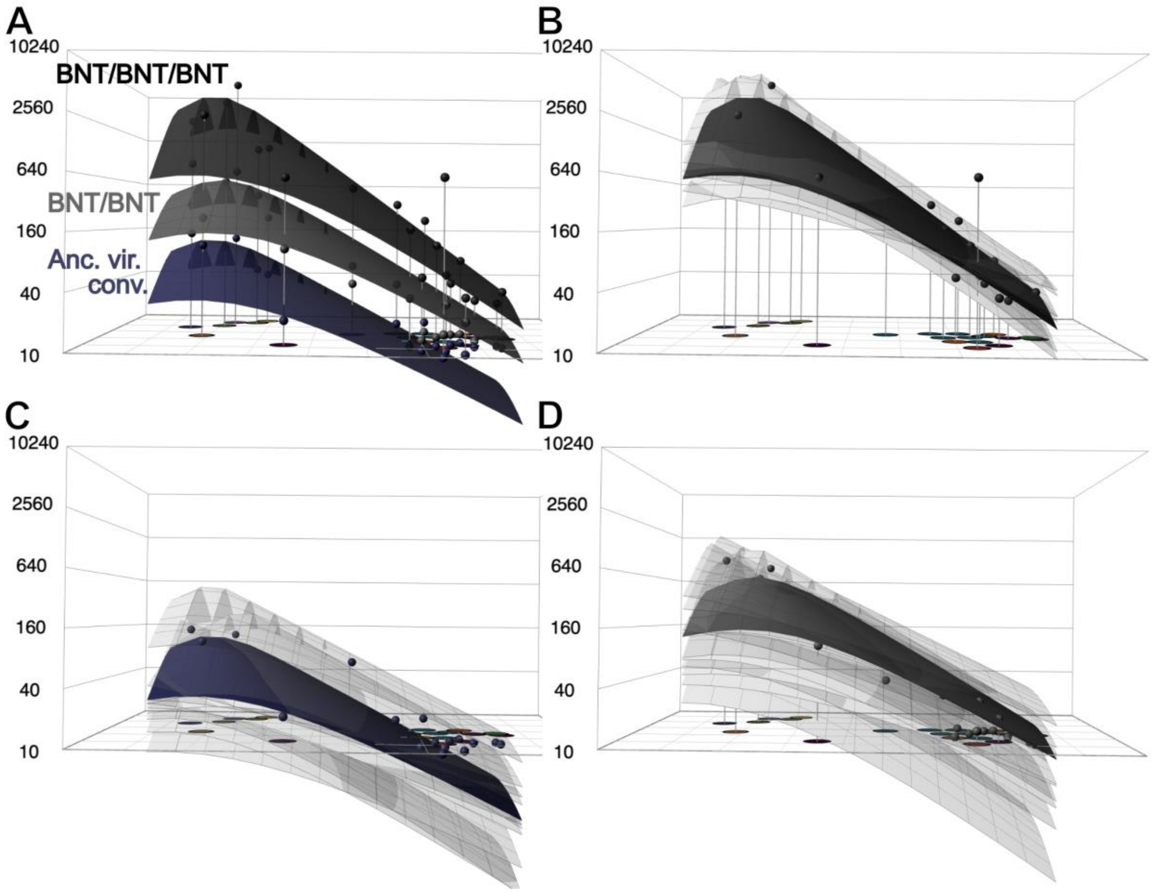

Supplementary Figure 23. Ancestral variant vaccinated or convalescant antibody landscapes. Antibody landscapes were fit for each serum in the different serum cohorts. Individual landscapes are shown in B-D as grey transparent surfaces, the geometric mean titer (GMT) landscape of each vaccine cohort is shown as fully opaque surface. GMTs against each variant are represented by small circles above the corresponding variant. The antibody landscapes were fit as described in the methods section. (A) GMT landscapes for triple BNT162b2 vaccinated (BNT/BNT/BNT), twice BNT162b2 vaccinated (BNT/BNT) and single ancestral virus infected individuals. Individual landscapes and GMT landscapes for BNT/BNT/BNT (B), Ancestral virus convalescent (C), BNT/BNT (D).

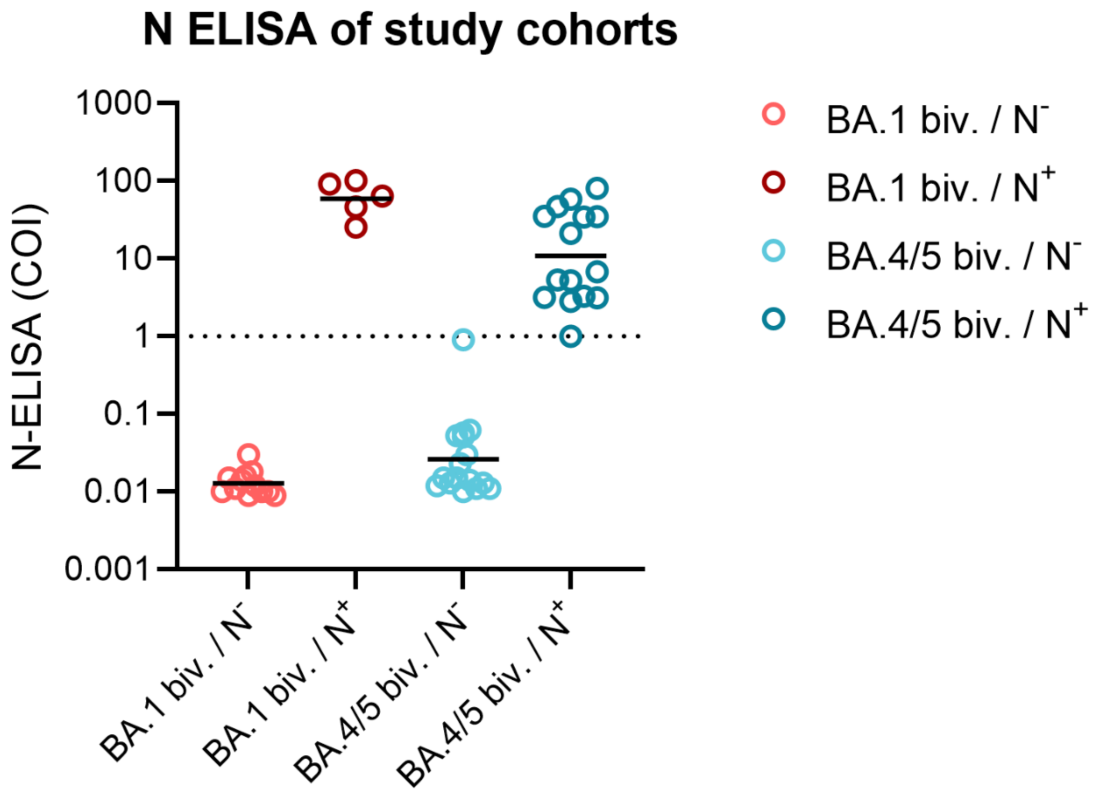

Supplementary Figure 24. Grouping of cohorts according to anti-nucleocapsid antibodies. Plasma samples were tested for anti-nucleocapsid (N) antibodies using the Elecsys Anti-N assay by Roche. Cut-off index (COI) ≥ 1 (dotted line) were treated as positive and samples were grouped accordingly into N negative (without infection history) and N positive (with previous SARS-CoV-2 infection) individuals. Shown are individual values (n=12 for ancestral + BA.1 boost without detectable N antibodies (BA.1 biv./N^-), n=5 for ancestral + BA.1 boost with positive N ELISA (BA.1 biv./N^+), n=16 for ancestral + BA.4/5 boost without detectable N antibodies (BA.4/5 biv./N^-), n=15 for ancestral + BA.4/5 boost with positive N ELISA (BA.4/5 biv./N^+)) and mean.

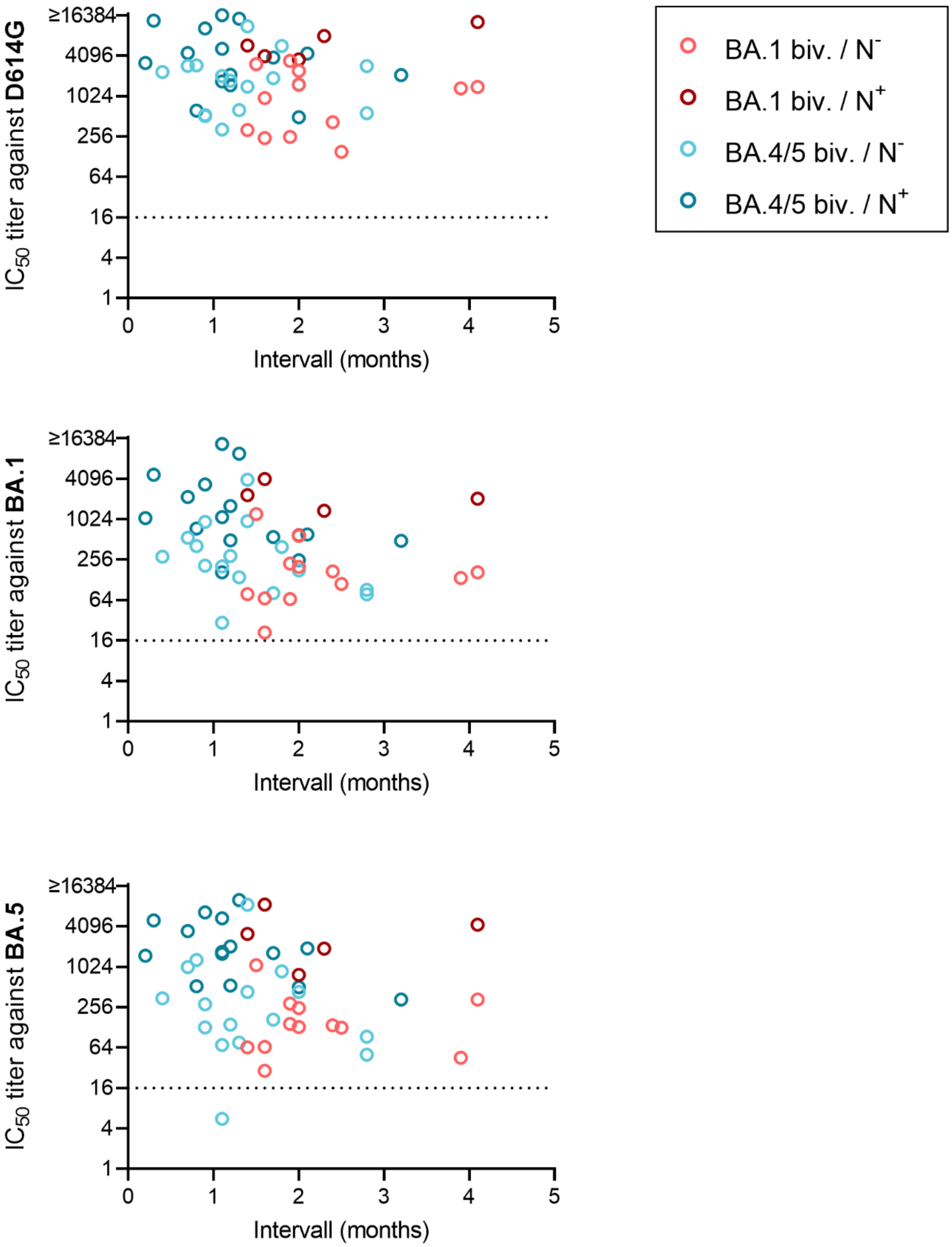

Supplementary Figure 25. Neutralizing antibodies relative to interval between bivalent booster and blood collection. Titers of neutralizing antibodies against D614G, BA.1 and BA.5 were blotted against the interval between bivalent booster and blood collection. IC_{50} titers below 16 were treated as negative (indicated by dotted lines). IC_{50} titer = 50% neutralization titer, N = nucleocapsid, BA.1 biv./N^- = ancestral + BA.1 boost without detectable N antibodies, BA.1 biv./N^+ = ancestral + BA.1 boost with positive N ELISA, BA.4/5 biv./N^- = ancestral + BA.4/5 boost without detectable N antibodies, BA.4/5 biv./N^+ = ancestral + BA.4/5 boost with positive N ELISA.

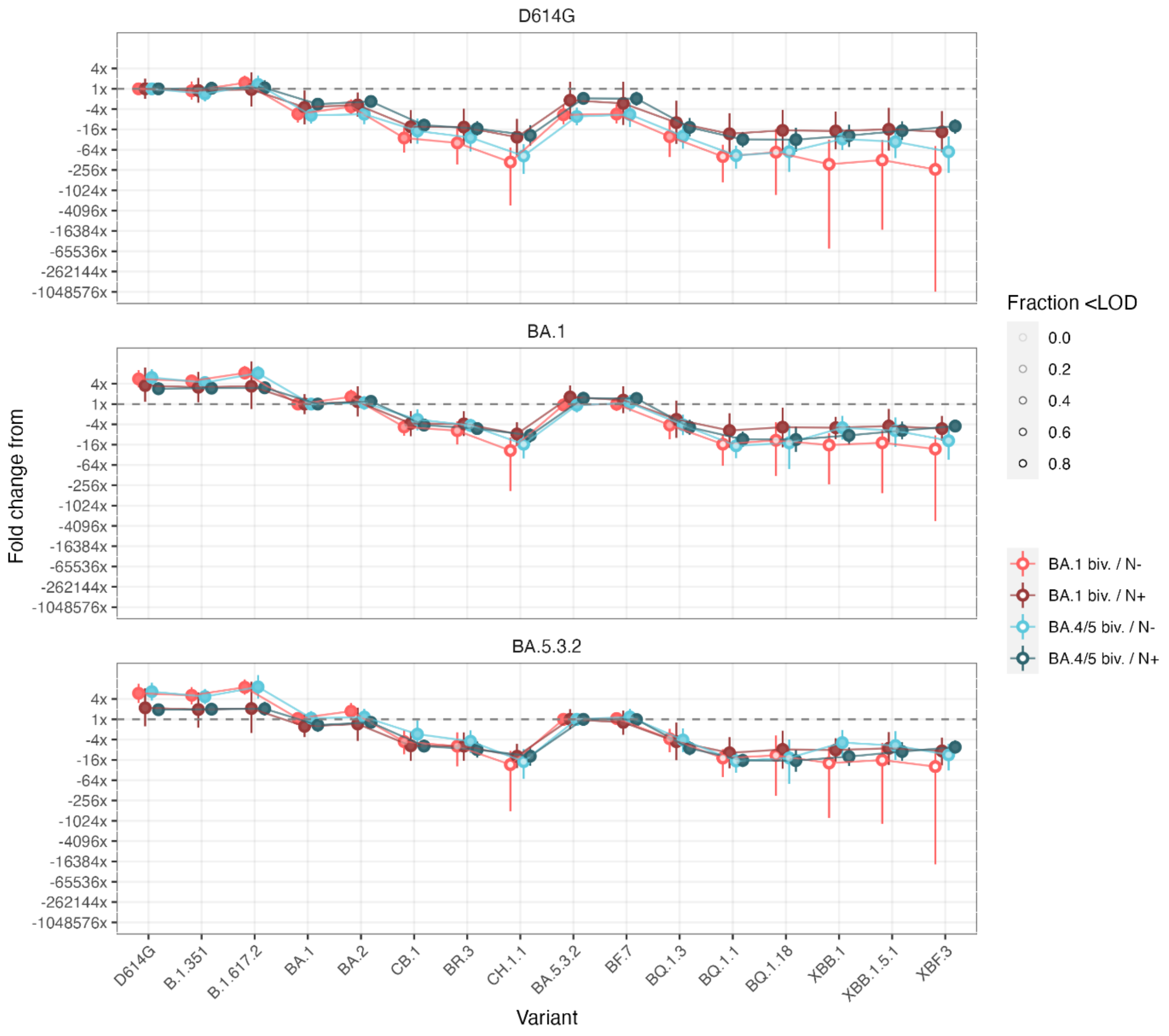

Supplementary Figure 26. Titer fold changes for bivalent vaccine groups. Mean fold changes from D614G (top graph), BA.1 (middle graph) or BA.5.3.2 (lower graph) to variants indicated on the x axis and 95% CI (confidence interval) were calculated for each bivalent vaccine group (n=12 for ancestral + BA.1 boost without detectable N antibodies (BA.1 biv./N^-), n=5 for ancestral + BA.1 boost with positive N ELISA (BA.1 biv./N^+), n=16 for ancestral + BA.4/5 boost without detectable N antibodies (BA.4/5 biv./N^-), n=15 for ancestral + BA.4/5 boost with positive N ELISA (BA.4/5 biv./N^+)) using the titertools R package,[1] where below threshold values are interpolated using a Bayesian approach. The whiteness of each point corresponds to the fraction of titers <LOD, increasing with the number of samples <LOD. For fully colored circles, all samples had detectable titers against the respective variant. N = nucleocapsid, LOD = limit of detection.

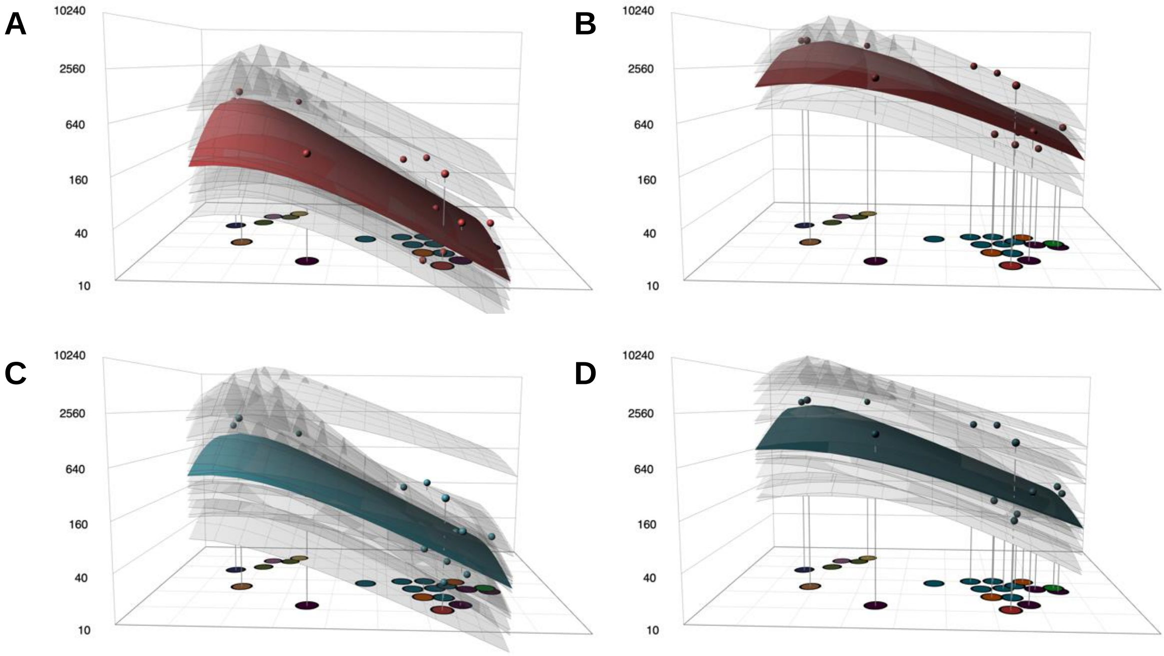

Supplementary Figure 27. Individual antibody landscapes. Antibody landscapes were fit for each serum in the different bivalent vaccine cohorts. Individual landscapes are shown as grey transparent surfaces, the geometric mean titer (GMT) landscape of each vaccine cohort is shown as fully opaque surface, the GMTs against each variant are represented by small circles above the corresponding variant. The antibody landscapes were fit as described in the methods section. (A) BA.1 biv. / N^-, (B) BA.1 biv. / N^+, (C) BA.4/5 biv. / N^-, (D) BA.4/5 biv. / N^+.

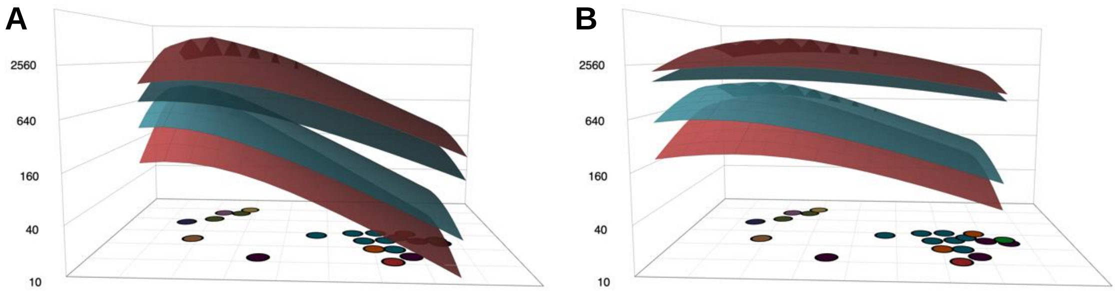

Supplementary Figure 28. Antibody landscapes fit to a subset of variants. Geometric mean titer (GMT) antibody landscapes were fit for the different bivalent vaccine cohorts to (A) pre-Omicron, early-Omicron and late-Omicron variants or (B) to pre-Omicron and early Omicron variants (excluding CB.1, BR.3, CH.1.1, BA.5.2.1, BE.1.1, BF.7, BQ.1.3, BQ.1.1, BQ.1.18, XBB.1, XBB.1.5, XBF). The antibody landscapes were fit as described in the methods section and the colors encode the bivalent vaccine cohort (red: BA.1 biv. / N^-, dark red: BA.1 biv. / N^+, turquoise: BA.4/5 biv. / N^-, dark turquoise: BA.4/5 biv. / N^+).

Supplementary Tables

Supplementary Table 1. Patient characteristics

					Anti-nucleocapsid ELISA		SARS-CoV-2 infection	
**Study cohort**	**Number of Participants**	**Mean age [years] ± SD**	**% female (number)**	**Mean interval between bivalent booster and blood collection [months] ± SD**	**Number positive**	**Mean COI ± SD**	**% with known infection history (number)**	**Variant#**
BA.1 biv./N^-	12	60.3 ± 10.9	50 (6)	2.2 ± 0.8	0	-	0	-
BA.1 biv./N^+	5	71.8 ± 10.8	60.0 (3)	2.3 ± 1.0	5	65.9 ± 28.2	0 (0)	-
BA.4/5 biv./N^-	16	66.4 ± 15.3	68.8 (11)	1.4 ± 0.7	0	-	-	-
BA.4/5 biv./N^+	15	62.6 ± 21.3	60.0 (9)	1.3 ±0.7	15	22.9 ± 24.0	37.5 (4)	alpha (n=1)<br>BA.2 Omicron (n=3)

n: number; COI: cut-off index, COI ≥ 1 was considered positive as specified by the manufacturer, N: nucleocapsid, BA.1 biv./N^-: ancestral + BA.1 boost without detectable N antibodies, BA.1 biv./N^+: ancestral + BA.1 boost with positive N ELISA, BA.4/5 biv./N^-: ancestral + BA.4/5 boost without detectable N antibodies, BA.4/5 biv./N^+: ancestral + BA.4/5 boost with positive N ELISA
#For two of the Omicron BA.2 convalescent individuals infecting virus variant was determined by sequencing or melting curve analysis; for one BA.2 and the alpha convalescent individual infecting virus variant was assumed based on time point of infection.

Supplementary Table 2. Virus variants used.

Isolate ID*	Pango lineage[§]	Category	GISAID ID
B86.2	B.1.177 (D614G; ancestral)	pre-Omicron	EPI_ISL_3305837
C63.1	B.1.1.7	pre-Omicron	EPI_ISL_3277382
C79.2	B.1.1.7 (E484K)	pre-Omicron	EPI_ISL_3277383
C24.1	B.1.351	pre-Omicron	EPI_ISL_17528983
D94	P.1.1	pre-Omicron	EPI_ISL_2095177
D27	B.1.617.2	pre-Omicron	EPI_ISL_2290769
E16.1	BA.1	BA.1 Omicron	EPI_ISL_17528984
E65.1	BA.2	BA.2 Omicron	EPI_ISL_12486408
F14.2	CB.1	BA.2 Omicron (BA.2.75)	EPI_ISL_16679179
F69.1	BR.3	BA.2 Omicron (BA.2.75)	EPI_ISL_17076114
G19.2	CH.1.1	BA.2 Omicron (BA.2.75)	EPI_ISL_16744347
E73.1	BA.5.3.2	BA.5 Omicron	EPI_ISL_13666092
F05.2	BA.5.2.1	BA.5 Omicron	EPI_ISL_17528948
F11.2	BE.1.1	BA.5 Omicron	EPI_ISL_17528949
F96.2	BF.7	BA.5 Omicron	EPI_ISL_16744346
F26.1	BQ.1.3	BA.5 Omicron	EPI_ISL_17528950
F74.1	BQ.1.1	BA.5 Omicron	EPI_ISL_17076149
F80	BQ.1.18	BA.5 Omicron	EPI_ISL_17077092
G22.3	XBB.1	recombinant	EPI_ISL_17076150
G37.2	XBB.1.5.1	recombinant	EPI_ISL_17077093
G45.1	XBF.3	recombinant	EPI_ISL_17324524

*Internal name of isolate; [§]determined using UShER (https://genome.ucsc.edu/cgi-bin/hgPhyloPlace) on 24.03.2023

Supplementary Table 3. Overview on sera used for calculation of the antigenic map.*

Variant	mR1273/ mR1273	BNT/BNT	AZ/BNT	AZ/AZ	Anc. virus conv.	alpha/alpha+ E484K conv.	beta conv.	delta conv.	BA.1 conv.	BA.2 conv.	BA.5 conv.	CK.2.1.1 conv.
D614G	10	11	10	10	10	9	6	5	14	10	1	2
B.1.1.7	10	11	10	10	10	9	6	5	14	10	-	-
B.1.1.7+E484K	10	11	10	10	10	9	6	5	14	10	-	-
B.1.351	10	11	10	10	10	9	6	5	14	10	1	2
P.1.1	10	11	10	10	10	9	6	5	14	10	-	-
B.1.617.2	10	11	10	10	10	9	6	5	14	10	1	2
BA.1	10	11	10	10	10	9	6	5	14	10	1	2
BA.2	10	11	10	10	10	9	6	5	14	10	1	2
CB.1	-	6	-	-	5	9	3	5	13	10	1	2
BR.3	-	6	-	-	5	9	3	5	13	10	1	2
CH.1.1	-	6	-	-	5	9	3	5	13	10	1	2
BA.5.3.2	10	11	10	10	10	9	6	5	14	10	1	2
BA.5.2.1	-	6	-	-	5	9	3	5	13	10	1	2
BE.1.1	-	6	-	-	5	9	3	5	13	10	1	2
BF.7	-	6	-	-	5	9	3	5	13	10	1	2
BQ.1.3	-	6	-	-	5	9	3	5	13	10	1	2
BQ.1.1	-	6	-	-	5	9	3	5	13	10	1	2
BQ.1.18	-	6	-	-	5	9	3	5	13	10	1	2
XBB.1	-	6	-	-	5	9	3	5	13	10	1	2
XBB.1.5	-	6	-	-	5	9	3	5	13	10	1	2
XBF.3	-	6	-	-	5	9	3	5	13	10	1	2

*A previous map was extended to include more recent variants of concern and BA.5 and CK.2.1.1 conv. (= convalescent) sera. Not all sera that were used to construct the previous map were titrated against the additional variants due to low volume.

Supplementary Table 4. Primers for sequencing of SARS-CoV-2*

Name	Sequence
nCoV-2019_29_RIGHT	CAATGCTAGGGAGAGCTGCCTA
nCoV-2019_28_RIGHT	GCCAACAACAACAAGGCCAAAC
nCoV-2019_29_LEFT	TGAGGGAGCCTTGAATACACCA
nCoV-2019_27_RIGHT	ACTTGTCACGCCTAAACGAACA
nCoV-2019_28_LEFT	TTTGTGCTTTTTAGCCTTTCTGCT
nCoV-2019_26_RIGHT	GTACGCGTTCCATGTGGTCATT
nCoV-2019_27_LEFT	TGGATCACCGGTGGAATTGCTA
nCoV-2019_25_RIGHT	GGCTTTGGCTTTGCTGGAAATG
nCoV-2019_26_LEFT	GCCTTGAAGCCCCTTTTCTCTA
nCoV-2019_24_RIGHT	CTTCCCTCAGTCAGCACCTCAT
nCoV-2019_25_LEFT	TGCTGCTACTAAAATGTCAGAGTGT
nCoV-2019_23_RIGHT	GGGCACGTAGTGTAGCTAGTCA
nCoV-2019_24_LEFT	GCTGAACATGTCAACAACTCATATGA
nCoV-2019_22_RIGHT	CGCCACCAGATTTGCATCTGTT
nCoV-2019_23_LEFT	ACTTTAGAGTCCAACCAACAGAATCT
nCoV-2019_21_RIGHT	TTACAACCAGAACTCAATTACCCCCTGCATACAC
nCoV-2019_22_LEFT	GTGATGTTCTTGTTAACAACTAAACGAACA
nCoV-2019_20_RIGHT	CCGGGTGTTGCTATGCCTAATC
nCoV-2019_21_LEFT	TCTGTAGTTTCTAAGGTTGTCAAAGTGA
nCoV-2019_19_RIGHT	GGGACACTTTGATGGACAACAGG
nCoV-2019_20_LEFT	ACAATTTGATACTTATAACCTCTGGAACAC
nCoV-2019_18_RIGHT	TGCTTTTCCACTGCTTCAGACA
nCoV-2019_19_LEFT	GGCACATGGCTTTGAGTTGACA
nCoV-2019_17_RIGHT	CCTTACACGTAACCCTGCTTGG
nCoV-2019_18_LEFT	GCACATAAAGACAAATCAGCTCAATGC
nCoV-2019_16_RIGHT	CGTGAAGTGCTGTCTGACAGAG
nCoV-2019_17_LEFT	TCAAGCTTTTTGCAGCAGAAACG
nCoV-2019_15_RIGHT	CTCTGACGATGCTGTTGTGTGT
nCoV-2019_16_LEFT	ACAACACAGACTTTATGAGTGTCTCT
nCoV-2019_14_RIGHT	ACGTGCTTTTCAGTAGCTGCAC
nCoV-2019_15_LEFT	TTTTAAGGAATTACTTGTGTATGCTGCT
nCoV-2019_13_RIGHT	TGTCGCTTCCAAGAAAAGGACG
nCoV-2019_14_LEFT	ACAGGCACTAGTACTGATGTCGT
nCoV-2019_12_RIGHT	GCATCAGCATTGTGGGAAATCC
nCoV-2019_13_LEFT	ACCTCTTACAACAGCAGCCAAAC
nCoV-2019_11_RIGHT	ATCAAGCCATTTCCATGTGGGC
nCoV-2019_12_LEFT	ATGGTGCTAGGAGAGTGTGGAC
nCoV-2019_10_RIGHT	TACCAATGTGCTATGAGGCCCA
nCoV-2019_11_LEFT	AGACACCTAAGTATAAGTTTGTTCGCA
nCoV-2019_9_RIGHT	TGGTGCTTTGGACATATCAGCA
nCoV-2019_10_LEFT	TTTACCAGGAGTTTTCTGTGGTGT
nCoV-2019_8_RIGHT	ATGCGCAGGTAGCAAAAGTCA

nCoV-2019_9_LEFT	AGAAGTTACTGGCGATAGTTGTAATAACT
nCoV-2019_7_RIGHT	TACAAATGGCCCCGATTTCAGC
nCoV-2019_8_LEFT	CAATCATGCAATTGTTTTTCAGCTATTTTG
nCoV-2019_6_RIGHT	GACGCGCAGGGAATGGATAATC
nCoV-2019_7_LEFT	ACCTGGTGTATACGTTGTCTTTGG
nCoV-2019_5_RIGHT	TCCACCTGCTCTACAAGATGCT
nCoV-2019_6_LEFT	ACTTCTATTAAATGGGCAGATAACAACTG
nCoV-2019_4_RIGHT	ATAACCACTTACCCGGGTCAGG
nCoV-2019_5_LEFT	ACCTACTAAAAAGGCTGGTGGC
nCoV-2019_3_RIGHT	CAAGACGGCAGTGAGGACAATC
nCoV-2019_4_LEFT	GGAATTTGGTGCCACTTCTGCT
nCoV-2019_2_RIGHT	CGGTGGACAAATTGTCACCTGT
nCoV-2019_3_LEFT	GGCTTGAAGAGAAGTTTAAGGAAGGT
nCoV-2019_1_RIGHT	GCGTCACCAAATGAATGCAACC
nCoV-2019_2_LEFT	CCATAATCAAGACTATTCAACCAAGGGT
nCoV-2019_1_LEFT	ACCAACCAACTTTCGATCTCTTGT

*Midnight-ONT/V3 primers as available at https://github.com/epi2me-labs/wf-artic/tree/master/data/primer_schemes/SARS-CoV-2/Midnight-ONT/V3

Supplementary References

1 Wilks, S. *Titertools: A statistical toolkit for the annalysis of censored titration data_. R package version 0.0.0.9001*.

2 Rössler, A., Riepler, L., Bante, D., von Laer, D. & Kimpel, J. SARS-CoV-2 Omicron Variant Neutralization in Serum from Vaccinated and Convalescent Persons. *N Engl J Med*, doi:10.1056/NEJMc2119236 (2022).

3 Rössler, A. *et al.* BA.2 and BA.5 omicron differ immunologically from both BA.1 omicron and pre-omicron variants. *Nature Communications* **13**, 7701, doi:10.1038/s41467-022-35312-3 (2022).

4 Rössler, A., Knabl, L., von Laer, D. & Kimpel, J. Neutralization Profile after Recovery from SARS-CoV-2 Omicron Infection. *N Engl J Med*, doi:10.1056/NEJMc2201607 (2022).
