# Supplementary material for: Characterizing SARS-CoV-2 neutralization profiles after bivalent boosting using antigenic cartography
Source: Nat Commun. 2023 Aug 26;14:5224. doi: 10.1038/s41467-023-41049-4 (PMC10460376; doi:10.1038/s41467-023-41049-4)
Supplement: Supplementary file 3 — Reporting Summary [file 41467_2023_41049_MOESM3_ESM.pdf]

Corresponding author(s): Derek J. Smith & Janine Kimpel

Last updated by author(s): Aug 16, 2023

Reporting Summary

Nature Portfolio wishes to improve the reproducibility of the work that we publish. This form provides structure for consistency and transparency in reporting. For further information on Nature Portfolio policies, see our Editorial Policies and the Editorial Policy Checklist.

Statistics

For all statistical analyses, confirm that the following items are present in the figure legend, table legend, main text, or Methods section.

n/a	Confirmed	
☐	☒	The exact sample size (*n*) for each experimental group/condition, given as a discrete number and unit of measurement
☐	☒	A statement on whether measurements were taken from distinct samples or whether the same sample was measured repeatedly
☒	☐	The statistical test(s) used AND whether they are one- or two-sided<br>*Only common tests should be described solely by name; describe more complex techniques in the Methods section.*
☐	☒	A description of all covariates tested
☐	☒	A description of any assumptions or corrections, such as tests of normality and adjustment for multiple comparisons
☐	☒	A full description of the statistical parameters including central tendency (e.g. means) or other basic estimates (e.g. regression coefficient) AND variation (e.g. standard deviation) or associated estimates of uncertainty (e.g. confidence intervals)
☒	☐	For null hypothesis testing, the test statistic (e.g. *F*, *t*, *r*) with confidence intervals, effect sizes, degrees of freedom and *P* value noted<br>*Give P values as exact values whenever suitable.*
☐	☒	For Bayesian analysis, information on the choice of priors and Markov chain Monte Carlo settings
☒	☐	For hierarchical and complex designs, identification of the appropriate level for tests and full reporting of outcomes
☒	☐	Estimates of effect sizes (e.g. Cohen's *d*, Pearson's *r*), indicating how they were calculated

Our web collection on statistics for biologists contains articles on many of the points above.

Software and code

Policy information about availability of computer code

Data collection	Fluorescent spots for neutralization assays were counted with an ImmunoSpot S6 Ultra-V reader and CTL analyzer BioSpot® 5.0 software (CTL Europe GmbH, Bonn, Germany). Calculation of 50% neutralizing antibody titers and statistical significance was performed in Graphpad prism version 9.0.1 as described in the Methods Methods section. The code for the antigenic cartography is publicly available as GitHub repository acorg/roessler_netzl_et_al2023 (https://github.com/acorg/roessler_netzl_et_al2023.git). Antigenic maps and antibody landscapes were made in R version 4.2.2 using the Racmacs (version 1.1.35) and ablandscapes (version 1.1.0) packages. Geometric mean titers (GMTs) for the multi-exposure groups and fold -change calculation were performed using the titertools package version 0.0.0.9001. All packages are free and open source available on GitHub and linked in the manuscript's GitHub repository. Sequencing data was aquired using a R9.4.1 flowcells in an Mk1B sequencer (ONT) and Ubuntu 20.04 with MinKNOW (v22.05.5 – v22.12.7, ONT).
Data analysis	Calculation of 50% neutralizing antibody titers and statistical significance was performed in Graphpad prism version 9.0.1 as described in the Methods Methods section. The code for the antigenic cartography is publicly available as GitHub repository acorg/roessler_netzl_et_al2023 (https://github.com/acorg/roessler_netzl_et_al2023.git). Antigenic maps and antibody landscapes were made in R version 4.2.2 using the Racmacs (version 1.1.35) and ablandscapes (version 1.1.0) packages. Geometric mean titers (GMTs) for the multi-exposure groups and fold -change calculation were performed using the titertools package version 0.0.0.9001. All packages are free and open source available on GitHub and linked in the manuscript's GitHub repository.<br>Pango lineage of SARS-CoV-2 isolates were determined using UShER (https://genome.ucsc.edu/cgi-bin/hgPhyloPlace) on 24.03.2023. Graphics depicting spike mutations relative to Wuhan-1 (Supplementary Figures 1-5, 21) were generated using https://covdb.stanford.edu/sierra/sars2/by-sequences/.<br>For sequencing, following data acquisition on a workstation running Ubuntu 20.04 with MinKNOW (v22.05.5 – v22.12.7, ONT), raw read fast5 files were converted to adapter- and barcode-trimmed fastq files, filtered to phred quality scoreQ10, using the super high accuracy model of Guppy (ONT, v6.1.5 – v6.4.6). The ARTIC Network pipeline for SARS-CoV-2 was pulled from https://github.com/epi2me-labs/wf-artic and

run using Nextflow (v22.04.4). Here, sequencing reads were filtered to a length between 200 and 1200 bp, aligned to the SARS CoV 2 reference sequence MN908947.3 using the map-ont preset of minimap2 (v2.18) [https://doi.org/10.1093/bioinformatics/bty191 , https://doi.org/10.1093/bioinformatics/btab705], primer sequences soft-trimmed, and resulting bam-files sorted and indexed using samtools (v1.12) [https://doi.org/10.1093/gigascience/giab008]. Variant calling used medaka (ONT, v1.5.0) with the r941_min_hac_variant_g507 model. Finally, a consensus sequence was generated using the bcftools consensus module (v1.12) [https://doi.org/10.1093/gigascience/giab008] and saved as a FASTA file.

For manuscripts utilizing custom algorithms or software that are central to the research but not yet described in published literature, software must be made available to editors and reviewers. We strongly encourage code deposition in a community repository (e.g. GitHub). See the Nature Portfolio guidelines for submitting code & software for further information.

Data

Policy information about availability of data

All manuscripts must include a data availability statement. This statement should provide the following information, where applicable:

- Accession codes, unique identifiers, or web links for publicly available datasets
- A description of any restrictions on data availability
- For clinical datasets or third party data, please ensure that the statement adheres to our policy

Data is available as GitHub repository acorg/roessler_netzl_et_al2023 (https://doi.org/10.5281/zenodo.7341691). Pango lineage of SARS-CoV-2 isolates were determined using UShER (https://genome.ucsc.edu/cgi-bin/hgPhyloPlace) on 24.03.2023. Graphics depicting spike mutations relative to Wuhan-1 (Supplementary Figures 1-5, 21) were generated using https://covdb.stanford.edu/sierra/sars2/by-sequences/. Sequences of SARS-CoV-2 variants used in this study are available at GISAID (see Supplementary Table 2 for GISAID IDs).

Research involving human participants, their data, or biological material

Policy information about studies with human participants or human data. See also policy information about sex, gender (identity/presentation), and sexual orientation and race, ethnicity and racism.

Reporting on sex and gender	Number and percentage of female participants (self-reported) for each of the groups analyzed are given in Supplementary Table 1. However, no information on individual participant level is given.<br>Differences in neutralizing antibody titers between male and female participants were not analyzed due to small group sizes. Additionally, a number of previous studies report no differences in antibody titers between male and female study participants.
Reporting on race, ethnicity, or other socially relevant groupings	No information on race, ethnicity etc. was collected from study participants.
Population characteristics	Participants with 4 doses of COVID-19 vaccination with or without SARS-CoV-2 infection history were included into this study. Patient characteristics are described in the Supplementary Materials in Table S1.
Recruitment	Individuals who had received a bivalent booster were invited to participate in the study. Participants after a fourth dose immunization with a bivalent booster, ancestral + BA.1 or ancestral + BA.4/5, were included into this study. Individuals who had received a bivalent booster were invited to participate in the study. No compensation for participation in the study was paid. Samples were included based on vaccination and/or infection status of participants. Participants provided information on vaccination status and previous infection. Sample selection might be biased due to limited sample size, however this bias should not influence the major conclusion of the study as relative changes of neutralizing antibodies within each individual patient were calculated for different SARS-CoV-2 variants.
Ethics oversight	The ethics committee (EC) of the Medical University of Innsbruck has approved the study with EC numbers: 1100/2020, 1111/2020, 1330/2020, 1064/2021, 1093/2021, 1168/2021, 1191/2021, 1197/2021, and 1059/2022. Informed consent has been obtained from study participants.

Note that full information on the approval of the study protocol must also be provided in the manuscript.

Field-specific reporting

Please select the one below that is the best fit for your research. If you are not sure, read the appropriate sections before making your selection.

☒ Life sciences ☐ Behavioural & social sciences ☐ Ecological, evolutionary & environmental sciences

For a reference copy of the document with all sections, see nature.com/documents/nr-reporting-summary-flat.pdf

Life sciences study design

All studies must disclose on these points even when the disclosure is negative.

Sample size	No sample size calculation was performed. We chose sample sizes based on previous studies (Rössler et al. 2022, Nature Communications 13, 7701). Additionally, we performed map diagnosis as described in the Supplementary Materials to ensure robustness of the generated antigenic maps.

Data exclusions	One sample in the BA.1 biv./N- group had been excluded from the analysis as it did not show neutralization of any of the analyzed virus variants.
Replication	We previously decribed the reproducibility of the here used neutralization assay (Riepler et al. 2020, Vaccines (Basel) 9). Each sample was analyzed once in the assay and experiments were not replicated. For antigenic mapping, robustness of maps has been analyzed as detailed in the Supplementary Materials, e.g. by exclusion of sera or virus variants or by introducing noice to the data.
Randomization	Not applicable as participants were allocated to the different groups due their COVID-19 vaccination regimens, which they received outside the study, and their SARS-CoV-2 infection history based on anti-Nucleocapsid antibodies.
Blinding	No blinding of samples during analysis in neutralization assays was performed. However, the number of infected cells in the neutralization assay were automatically counted using an immunospot reader removing potential bias in data aquisition. Additionally, manual quality control to remove fibres etc. has been done blinded. IC50 titers were calculated automatically using Graphpad Prism.

Reporting for specific materials, systems and methods

We require information from authors about some types of materials, experimental systems and methods used in many studies. Here, indicate whether each material, system or method listed is relevant to your study. If you are not sure if a list item applies to your research, read the appropriate section before selecting a response.

Materials & experimental systems

n/a	Involved in the study	
☐	☒	Antibodies
☐	☒	Eukaryotic cell lines
☒	☐	Palaeontology and archaeology
☒	☐	Animals and other organisms
☒	☐	Clinical data
☒	☐	Dual use research of concern
☒	☐	Plants

Methods

n/a	Involved in the study	
☒	☐	ChIP-seq
☒	☐	Flow cytometry
☒	☐	MRI-based neuroimaging

Antibodies

Antibodies used	SARS-CoV-2 convalescent serum 1:1,000 diluted as primary antibody followed by goat anti-human Alexa Fluor Plus 488-conjugated secondary antibody, 1:1,000 diluted; Ref. A48276, Invitrogen, Thermo Fisher Scientific, Vienna, Austria
Validation	No antibody validation has been performed during this study. Primary antibody has been used and validated for a previous study (Riepler et al. 2020, Vaccines (Basel) 9). The secondary antibody is commercially available and binds according to manufacturers specification to human IgG, but does not bind to non-immunoglobulin human serum proteins or serum proteins/IgG from bovine, mouse, and rabbit (https://www.thermofisher.com/order/genome-database/dataSheetPdf?producttype=antibody&productsubtype=antibody_secondary&productId=A48276&version=326).

Eukaryotic cell lines

Policy information about cell lines and Sex and Gender in Research

Cell line source(s)	In-house generated Vero (African green monkey kidny cell line) derivate stably overexpressing ACE2 and TMPRSS2
Authentication	no cell line authentication
Mycoplasma contamination	Cells were tested negative for mycoplasma
Commonly misidentified lines (See ICLAC register)	No commonly missidentified cell line has been used for this study.
